# Factors influencing the implementation of screening and brief interventions for alcohol use in primary care practices: a systematic review using the COM-B system and Theoretical Domains Framework

**DOI:** 10.1186/s13012-020-01073-0

**Published:** 2021-01-07

**Authors:** Frederico Rosário, Maria Inês Santos, Kathryn Angus, Leo Pas, Cristina Ribeiro, Niamh Fitzgerald

**Affiliations:** 1 Instituto de Medicina Preventiva e Saúde Pública, Faculty of Medicine, Lisbon University, Avenida Professor Egas Moniz, 1649-028 Lisbon, Portugal; 2 Agrupamento de Centros de Saúde Dão Lafões, Av. António José de Almeida - Edíficio MAS, 3514-511 Viseu, Portugal; 3 Hospital Casa de Saúde São Mateus SA, Rua 5 de Outubro 183, 3500-093 Viseu, Portugal; 4 Institute for Social Marketing & Health (ISMH), Faculty of Health Sciences & Sport, University of Stirling, Stirling, FK9 4LA Scotland, UK; 5 Academic Centre for General Practice, KU Leuven, Kapucijnenvoer 33 blok j - box 7001, 3000 Leuven, Belgium

**Keywords:** Alcohol-induced disorders, Screening, Counselling, Primary health care, Review (publication type), Psychological theory

## Abstract

**Background:**

Alcohol is a leading risk factor contributing to the global burden of disease. Several national and international agencies recommend that screening and brief interventions (SBI) should be routinely delivered in primary care settings to reducing patients’ alcohol consumption. However, evidence shows that such activities are seldom implemented in practice. A review of the barriers and facilitators mediating implementation, and how they fit with theoretical understandings of behaviour change, to inform the design of implementation interventions is lacking. This study aimed to conduct a theory-informed review of the factors influencing general practitioners’ and primary care nurses’ routine delivery of alcohol SBI in adults.

**Methods:**

A systematic literature search was carried out in four electronic databases (Medline, CINAHL, CENTRAL, PsycINFO) using comprehensive search strategies. Both qualitative and quantitative studies were included. Two authors independently abstracted and thematically grouped the data extracted. The barriers and facilitators identified were mapped to the domains of the Capability-Opportunity-Motivation-Behaviour system/Theoretical Domains Framework (TDF).

**Results:**

Eighty-four out of the 258 studies identified met the selection criteria. The majority of the studies reported data on the views of general practitioners (*n* = 60) and used a quantitative design (*n* = 49). A total of 660 data items pertaining to barriers and 253 data items pertaining to facilitators were extracted and thematically grouped into 46 themes. The themes mapped to at least one of the 14 domains of the TDF. The three TDF domains with the highest number of data units coded were ‘Environmental Context and Resources’ (*n* = 158, e.g. lack of time), ‘Beliefs about Capabilities’ (*n* = 134, e.g. beliefs about the ability to deliver screening and brief advice and in helping patients to cut down) and ‘Skills’ (*n* = 99, e.g. lack of training).

**Conclusions:**

This study identified a range of potential barriers and facilitators to the implementation of alcohol SBI delivery in primary care and adds to the scarce body of literature that identifies the barriers and facilitators from a theoretical perspective. Given that alcohol SBI is seldom implemented, this review provides researchers with a tool for designing novel theory-oriented interventions to support the implementation of such activity.

**Systematic review registration:**

PROSPERO CRD42016052681

**Supplementary Information:**

The online version contains supplementary material available at 10.1186/s13012-020-01073-0.

Contributions to the literature • Literature shows that the routine delivery of alcohol screening and brief interventions in primary health care has been hampered by barriers to implementation. Most implementation programmes in practice and research have lacked a theoretical rationale for how they would address these barriers.

• Our review is the first to analyse these barriers from a behavioural change point of view.

• Our review contributes to a better understanding of the barriers to implementation of alcohol screening and brief intervention in primary health care and provides researchers with a rationale for selecting the most promising actions to overcoming these barriers.

## Introduction

Alcohol misuse is a major risk factor for ill health and death [1], accountable for 5.3% of all deaths worldwide and 5.1% of the global burden of disease and injury [2]. The economic impact of alcohol use and related harm alone can reach as much as 3.3% of the Gross Domestic Product [3]. Even small reductions in alcohol intake can bring about significant health gains [4]. For example, a reduction in the daily average consumption of pure alcohol from 40 to 30 grammes (from 4 to 3 standard drinks) is associated with a 48% decrease in the risk of oral cancer, and a decrease in the risk of hypertension of 13% in men and 66% in women. The World Health Organization recommends the implementation of several high-impact strategies to change drinking behaviour, including the provision of alcohol screening and brief interventions (SBI) in primary health care settings [5].

In the past four decades, randomized controlled trials and meta-analyses have found alcohol SBI in primary care settings to be effective and cost-effective or cost-saving [6–13]. Alcohol increases the risk of several physical, mental and social conditions that present frequently in primary care [3, 4] and a significant proportion of patients visiting primary care drink least at a hazardous or higher level [14–16]. However, few at-risk drinkers are identified as such and counselled to cut down [17–23]. For example, a recent trial found that, prior to intervention, only 5.9% of the consulting patients were screened and, of the screen positives, 73.7% received advice [24]. Therefore, many at-risk drinkers leave their primary care appointment unaware of the risks of their alcohol consumption or how it might be contributing to current ill health. Notwithstanding recent debates questioning SBI effectiveness [25, 26], these represent missed opportunities to increase patients’ awareness of alcohol-related risks, a first step towards enabling them to make a more informed choice on whether or not to cut down [4].

Although there is a growing literature on barriers to and facilitators of the implementation of alcohol SBI in routine clinical practice, this information is scattered and provides an unclear representation of the factors affecting primary care providers’ systematic engagement with at-risk drinkers. A review by Johnson et al. identified the barriers to and facilitators of the delivery of screening and brief intervention for alcohol misuse [27] but prioritized studies judged to best inform UK practice and focused on several different healthcare settings. Lack of training, support from management and resources, as well as workload pressures were identified as the main barriers to implementation; whilst adequate resources, training and the identification of those at risk without stereotyping were the main facilitators. This review updates the Johnson et al. review, employs a more comprehensive search strategy and has an international focus.

Another gap in the evidence base is the lack of theoretical insights in this area [28]. Knowledge of how identified barriers and facilitators fit with the theoretical understandings of behaviour change can help in selecting the implementation interventions that have a higher chance of bringing about the desired change in practitioner behaviour. Our review is informed by the Capability-Opportunity-Motivation-Behaviour (COM-B) system [29] and Theoretical Domains Framework (TDF) [30] system in that the barriers and facilitators were mapped to the TDF domains which, in turn, fit with the COM-B system. The review aims to identify the theoretical concepts underpinning the barriers and facilitators to implementation. Our intention is to provide practical evidence for selecting the best strategies to increase the implementation of alcohol SBI in primary health care.

### Objectives

1. to identify barriers to and facilitators of routine delivery of alcohol screening and brief interventions in adults by general practitioners (GPs) and primary care nurses;
2. to review how the identified barriers and facilitators fit with theoretical understandings of behaviour change using the COM-B system and TDF framework.

## Methods

This review is reported according to the Preferred Reporting Items for Systematic Reviews and Meta-Analysis (PRISMA) statement (see Additional file 1) [31]. The protocol was pre-registered on PROSPERO (CRD42016052681) and published elsewhere [32]. No amendments were introduced to the review protocol.

### Information sources and searches

The following electronic databases were searched by KA, from onset of literature database until May 2016: MEDLINE, CINAHL, Cochrane Central Register of Controlled Trials (CENTRAL) and PsycINFO. The search combined terms for ‘Screening and Brief Interventions’, ‘Alcohol’ and ‘Primary Health Care’ (see Additional file 2).

### Eligibility criteria and study selection

To be included, a study had to:

- report primary data and be published in a peer-reviewed scientific journal;
- use a Delphi methodology, focus group, in-depth interview or semi-structured interview design for qualitative studies, or a randomized controlled trial, before-after with no control group, cohort, case-control or cross-sectional design for quantitative studies;
- address barriers and facilitators for implementing alcohol SBI reported by GPs or nurses working in primary care general practices (excluding out-of-hours practices or walk-in centres, full definition in protocol [32]);
- be available in full-text copy in English, French, Portuguese or Spanish.

Two reviewers (FR, MIS) independently screened the search results for relevant titles and abstracts. Full-text copies of studies meeting inclusion criteria and of those with unclear eligibility were sought and the screening process repeated by the same two reviewers. Disagreements were discussed and resolved by consensus.

### Data extraction and quality assessment

Data extraction was conducted independently by the two aforementioned reviewers and included the following: first author; publication year; title; country; language; study objective; study design; sample (sampling strategy, type and number of care providers, response/attrition rate); barriers and facilitators; main results; relation with outcomes or process variables in intervention studies.

The methodological quality of each study was independently assessed by two reviewers: half of the studies were appraised by FR and LP, the other half by FR and MIS. Disagreements were resolved through consensus. Quantitative studies were appraised with the NIH National Heart, Lung, and Blood Institute tools [33] and qualitative studies with the critical appraisal skills programme (CASP) qualitative research checklist [34]. The quality of the studies was further appraised as recommended by the Cochrane Collaboration Qualitative Methods Group [35]. Inclusion of studies was not influenced by methodological quality. The quality rating for each study was allocated in line with the guidance in the relevant tool.

### Data synthesis

The data items extracted (i.e. barriers and facilitators, defined as factors that decrease (barriers) or increase (facilitators) the probability of the implementation of the intervention by general practitioners/family physicians or nurses working in primary care practices) were independently extracted by two reviewers (FR, MIS) into a Microsoft Excel sheet. Next, data were grouped thematically: the aforementioned reviewers read and re-read the data items, grouping similar/related items into iteratively developed themes. Each theme was analysed and mapped to the capability, opportunity and motivation components of the COM-B system and the 14 TDF domains, all of which fell into one of these three components. To ensure the theme mapped to the TDF domain, we further checked that the extracted data within each theme fitted with the domain content (i.e. the component constructs in each TDF domain); to remain mapped to the TDF domain, themes had to have at least one data item linked to a component construct. TDF domains were mapped to COM-B components as defined by Cane and colleagues [30]. Disagreements between the reviewers were resolved through consensus. The results are tabulated and a narrative synthesis of the findings is provided, structured around the themes of barriers and facilitators, the professional group and the components and domains of the COM-B system and TDF framework.

## Results

### Study selection

**Fig. 1:**
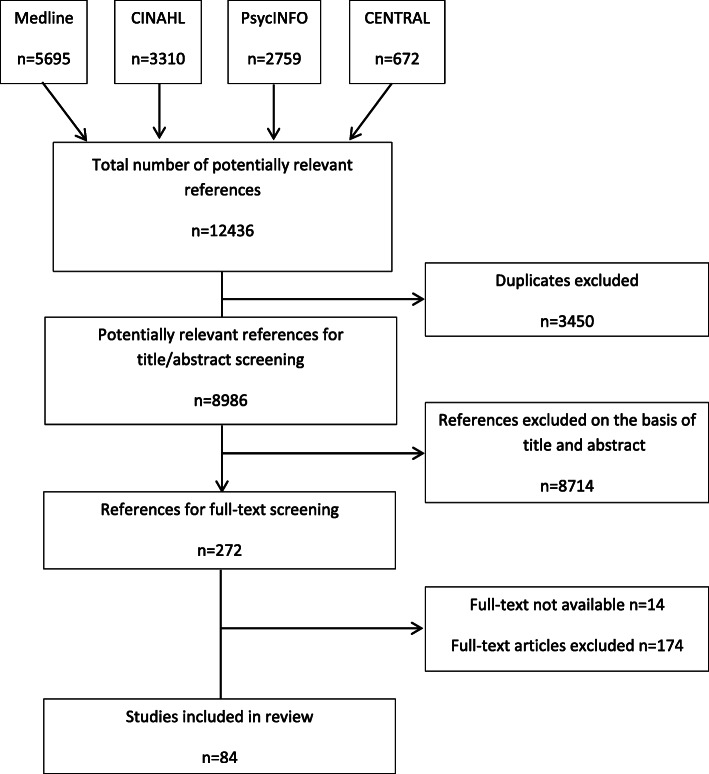
Flow diagram of screening process

The search strategy found 12,436 potentially relevant references (Fig. 1).

After duplicate removal, 8,986 unique references proceeded to abstract screening, from which 272 references were selected for full-text examination. We were unable to obtain full-text copies of 14 references (Additional file 3). Of the 258 remaining references, 174 with full-text were excluded (Additional file 4). Eighty-four studies published between 1982 and 2016 satisfied our criteria [16, 20, 23, 36–116] (Table 1). Of the studies included, 76 were single-site studies, mainly from Europe (*n* = 47), North America (*n* = 12) and Oceania (*n* = 12). Seventy-nine references were published in English, 3 in Portuguese and 2 in Spanish. Forty-nine studies were quantitative, mostly using a cross-sectional design; 30 were qualitative, mainly using focus groups and/or semi-structured interviews; the remaining 5 used a mixed-methods approach. Sixty studies reported data on GPs, 9 on nurses and 15 on both.

**Table 1:** Characteristics of included studies

First author	Year	Country	Language	Study design	Study sample (***n***)		Methodological quality
					GP	Nurse	
Aalto [36]	2001	Finland	English	Cross-sectional	84	167	Good
Aalto [38]	2003	Finland	English	Cross-sectional	64		Good
Aalto [37]	2003a	Finland	English	Focus group	18	19	Good
Abidi [39]	2016	Netherlands	English	Delphi	37		Good
Abouyanni [40]	2000	Australia	English	Cross-sectional	416		Poor
Aira [41]	2003	Finland	English	Semi-structured interviews	35		Good
Aira [42]	2004	Finland	English	Semi-structured interviews	35		Good
Ampt [43]	2009	Australia	English	Semi-structured interviews	15	1	Good
Anderson [44]	1985	UK	English	Cross-sectional	312		Good
Anderson [46]	2003	Australia, Belgium, Canada, France, Italy, New Zealand, Norway, Portugal, UK	English	Cross-sectional	1300		Good
Anderson [45]	2004	Australia, Belgium, Spain, UK	English	RCT	277		Good
Anderson [47]	2014	Czech Republic, Italy, Netherlands, Poland, Portugal, Spain, Slovenia, UK	English	Cross-sectional	2345		Fair
Arborelius [48]	1995	Sweden	English	Structured interviews	13		Fair
Beich [49]	2002	Denmark	English	Focus groups Individual interviews	24		Fair
Bendtsen [50]	2015	Netherlands, Poland, Spain, Sweden, UK	English	Cohort	409	282	Fair
Berner [51]	2007	Germany	English	Cross-sectional	58		Fair
Brennan [52]	2013	Australia	English	Cross-sectional	15		Poor
Brotons [53]	2005	Croatia, Estonia, Georgia, Greece, Ireland, Malta, Poland, Slovakia, Slovenia, Spain, Sweden	English	Cross-sectional	2082		Poor
Carlfjord [54]	2012	Sweden	English	Focus groups	9	12	Good
Casswell [55]	1982	New Zealand	English	Cross-sectional	431		Fair
Charrel [56]	2010	France	English	Cross-sectional	300		Fair
Clement [57]	1986	UK	English	Cross-sectional	71		Good
Clifford [58]	2011	Australia	English	Pre-post training surveys Focus groups	3	3	Good
Deehan [60]	1997	UK	English	Cross-sectional	81		Fair
Deehan [61]	1998	UK	English	Cross-sectional	2377		Poor
Deehan [59]	1999	UK	English	Cross-sectional	264	196	Fair
Farmer [62]	2001	UK	English	Semi-structured interviews Cross-sectional	50		Poor
Ferguson [63]	2003	USA	English	Cross-sectional	40		Poor
Fernández [64]	1999	Spain	Spanish	Cross-sectional	227		Fair
Friedmann [65]	2000	USA	English	Cross-sectional	243		Fair
Fucito [66]	2003	Australia	English	Cross-sectional	110		Good
Geirsson [20]	2005	Sweden	English	Cross-sectional	68	193	Good
Gurugama [67]	2003	Sri Lanka	English	Cross-sectional	105		Good
Haley [68]	2000	Canada	English	Cross-sectional	805		Fair
Harris [69]	2005	Australia	English	Pre-post questionnaire with no control group	21		Poor
Holmqvist [70]	2008	Sweden	English	Cross-sectional	1790	2549	Good
Hutchings [71]	2006	UK	English	Focus groups	18	15	Good
Johansson [73]	2002	Sweden	English	Cross-sectional	65	141	Good
Johansson [72]	2005	Sweden	English	Focus groups		26	Poor
Johansson [74]	2005a	Sweden	English	Focus groups	13		Good
Kaariainen [75]	2001	Finland	English	Cross-sectional	GP + nurse = 69		Fair
Kaner [78]	1999	UK	English	Cross-sectional	279		Good
Kaner [79]	2001	Australia, Belgium, Bulgaria, Canada, France, Hungary, Italy, New Zealand, Norway, Poland, Portugal, Thailand, UK	English	Cross-sectional	2139		Good
Kaner [76]	2003	UK	English	Cluster RCT		212 general practices	Fair
Kaner [77]	2006	UK	English	Interviews	29		Good
Kersnik [80]	2009	Slovenia	English	Focus groups	32		Good
Keurhorst [81]	2014	Netherlands	English	Cluster RCT	112		Fair
Kolsek [82]	2008	Belgium, Bulgaria, Hungary, Italy, Latvia, Russia, Slovenia	English	Delphi Focus groups	nr	nr	Fair
Koopman [83]	2008	South Africa	English	Cross-sectional	50		Fair
Lacey [84]	2009	UK	English	Focus groups Semi-structured interviews Cross-sectional		nr	Fair
Lambe [85]	2008	UK	English	Cross-sectional Focus groups		53	Good
Lid [86]	2012	Norway	English	Focus groups	13		Fair
Lid [87]	2015	Norway	English	Focus groups	19		Good
Linke [88]	2005	UK	English	Focus groups	10		Fair
Lock [89]	2002	UK	English	Semi-structured interviews		24	Good
Maheux [90]	1999	Canada	English	Cross-sectional	805		Fair
May [91]	2006	UK	English	Semi-structured interviews	43	1	Good
McAvoy [92]	2001	Australia, Canada, Denmark, France, Hungary, Italy, New Zealand, Norway, Poland, Russia	English	Semi-structured interviews	126		Fair
Miller [93]	2006	USA	English	Focus groups	nr	nr	Good
Miner [94]	1990	Spain	Spanish	Cross-sectional	83		Fair
Mistral [95]	2001	UK	English	Cross-sectional Semi-structured interviews	103		Poor
Moretti-Pires [96]	2011	Brazil	Portuguese	Focus groups Semi-structured interviews		12	Fair
Mules [97]	2012	New Zealand	English	Semi-structured interviews	19		Fair
Nevin [98]	2002	Canada	English	Cross-sectional	75		Fair
Nygaard [100]	2010	Norway	English	Cross-sectional	901		Good
Nygaard [99]	2011	Norway	English	Focus groups	40		Good
Owens [101]	2000	UK	English	Cross-sectional		101	Fair
Payne [102]	2005	Australia	English	Cross-sectional	170		Fair
Poplas Susic [103]	2010	Slovenia	English	Focus groups	32		Good
Proude [104]	2006	Australia	English	Pre-post questionnaire with no control group	300		Poor
Rapley [105]	2006	UK	English	Semi-structured interviews	43		Good
Ribeiro [16]	2011	Portugal	Portuguese	Cross-sectional	188		Fair
Richmond [106]	1998	Australia	English	Post-intervention questionnaire with no control group	272		Poor
Roche [107]	1991	Australia	English	Focus groups	44		Fair
Rush [108]	1994	Canada	English	Cross-sectional	1235		Good
Rush [109]	1995	Canada	English	Focus groups Semi-structured interviews	12		Good
Segnan [110]	1992	Italy	English	Cross-sectional	209		Fair
Sharp [111]	2011	USA	English	Cross-sectional	101		Good
Slaunwhite [112]	2015	Canada	English	Cross-sectional	67		Poor
Souza [113]	2012	Brazil	Portuguese	Semi-structured interviews		8	Fair
Van Zyl [114]	2013	South Africa	English	Cross-sectional	77		Fair
Vandermause [115]	2007	USA	English	In-depth interviews		23	Fair
Vinson [116]	2004	USA	English	Cluster RCT	44		Fair
Wilson [23]	2011	UK	English	Cross-sectional	282		Good

*nr* Not reported

### Methodological quality

We found considerable variation in the quality of the studies retained (Table 1). Of the 33 qualitative studies, 19 were considered to be good-, 12 fair- and 2 poor-quality studies [33–35]. Of the 51 quantitative studies, 18 were considered to be good-, 23 fair- and 10 poor-quality studies [33–35].

### Summary of findings

A total of 660 data items (descriptions or reports) pertaining to barriers were extracted. A total of 46 themes were identified from these data items (Table 2).

**Table 2:** TDF domains

COM-B component—TDF domain	Theme name	Definition of the theme	Study type—no survey/interview/focus group/mixed methods/other	No. of data items
				Barriers/facilitators
Capability—knowledge	Alcohol-related knowledge	Doctors’ and nurses’ knowledge about specific concepts related to alcohol screening and brief interventions (e.g. drinking limits, definition of heavy drinking, guidelines, screening questionnaires, content of brief interventions)	26/6/5/2/2	58/19
	Disease model training	An approach to the patient in that health providers ask about alcohol only when the patient present with specific symptoms and/or signs	2/2/1/0/0	5/0
	Patients’ receptiveness to alcohol interventions	The extent to which doctors and nurses think patients are open to be asked and advised about their drinking	1/3/1/1/0	3/4
	Doctors and nurses own drinking habits	The use of doctors and nurses own drinking behaviour as a benchmark to define whether or not a patient drinks excessively	0/2/1/0/0	3/0
	Alcohol being perceived as having health benefits	The extent to which doctors and nurses believe that drinking moderately improves health in general	2/1/0/0/0	3/0
	Knowledge of support services	Doctors’ and nurses’ knowledge of alcohol services where they could refer the patient to	2/0/0/0/0	2/0
Capability—skills	Training	The extent to which doctors and nurses agree they have received/need training in screening and advising at-risk drinkers	28/4/2/3/3	51/25
	Role adequacy	The extent to which doctors and nurses believe they have sufficient knowledge and skills to manage drinkers	16/4/2/3/4	45/0
	Demographical characteristics of the PHC professionals	Doctors’ and nurses’ demographical characteristics influencing their screening and advice performance	2/0/0/0/0	3/0
Capability—memory, attention and decision processes	Demographical characteristics of the patient	Patients’ demographical characteristics influencing doctors’ and nurses’ screening and advice performance	1/3/1/0/0	6/0
	Feedback on the results of delivering SBI	Information about doctors’ and nurses’ performance concerning screening, advice and/or effectiveness of their actions	0/1/1/0/1	1/2
	Remembering	Doctors’ and nurses’ perception of how easy/difficult it is to remember to ask about alcohol	0/1/1/0/0	2/0
Capability—behaviour regulation	Organization for preventive counselling	Doctors’ and nurses’ perception of the presence or absence of organization/systematic strategies to implement alcohol screening and brief advice	5/4/7/0/2	6/19
Motivation—beliefs about capabilities	Beliefs about the ability to deliver SBI and in helping patients to cut down	Doctors’ and nurses’ beliefs about, and/or confidence in, the effectiveness of their skills to screen and advise patients to reduce their alcohol intake	23/5/4/2/6	60/6
	Time	Time-related factors doctors and nurses believe to affect their capability to implement alcohol screening and brief interventions	9/7/10/3/3	31/14
	Difficult task	Difficulties perceived by doctors and nurses when asking and advising patients about alcohol	13/6/6/2/4	30/24
	Therapeutic commitment	Doctors’ and nurses’ predisposition to working therapeutically with people who have excessive alcohol consumption	3/0/0/0/2	5/0
	Self-esteem when working with at-risk drinkers	Doctors’ and nurses’ perceived self-worth when working with at-risk drinkers	4/0/1/0/0	4/3
	Disease model training	An approach to the patient in that health providers ask about alcohol only when the patient present with specific symptoms and/or signs	0/1/0/0/0	1/0
	Patients’ beliefs about alcohol	Doctors’ and nurses’ perceptions of the conceptions patients have about the effects of alcohol, either beneficial or detrimental	0/1/0/0/0	1/0
	Demographical characteristics of the patient	Patients’ demographical characteristics influencing doctors’ and nurses’ screening and advice performance	0/1/0/0/0	2/0
Motivation—beliefs about consequences	Effectiveness of SBI	Doctors’ and nurses’ beliefs about the effectiveness of asking and advising patients about their alcohol consumption	13/3/4/1/4	24/14
	Patients’ feelings when asked about their drinking	Doctors’ and nurses’ beliefs about how patients would feel if asked and advised about alcohol	5/6/6/1/0	22/3
	Therapeutic relation with the patient	The therapeutic alliance that is established between a healthcare professional and a patient	1/4/5/1/2	12/4
	Reliability of the answers of the patients when asked about alcohol	The degree to which doctors and nurses believe in the accuracy of the answers provided by patients concerning their alcohol consumption	1/5/2/0/1	9/0
	Patients’ receptiveness to alcohol interventions	The extent to which doctors and nurses think patients are open to be asked and advised about their drinking	4/3/2/1/0	7/4
	Patients’ reactions when asked about alcohol	Doctors’ and nurses’ beliefs about how patients would react if asked and advised about alcohol	3/4/0/2/1	7/3
	Frustrating task	Doctors’ and nurses’ beliefs about how they would feel if they were to implement alcohol screening and brief interventions	2/2/0/1/1	5/1
	Alcohol being perceived as having health benefits	The extent to which doctors and nurses believe that drinking moderately improves health in general	2/1/0/0/0	3/0
	Incentives	Doctors’ and nurses’ beliefs about what they would gain by implementing alcohol screening and brief interventions	4/1/2/1/1	2/22
	Time	Time-related factors doctors and nurses believe to affect their capability to implement alcohol screening and brief interventions	1/4/4/0/1	2/14
	Delivering SBI can make other patients suffer	Doctors’ and nurses’ belief that implementing alcohol screening and brief interventions could harm other patients	1/0/0/1/0	2/0
	Bad publicity	Doctors’ and nurses’ belief that dealing with at-risk drinkers could give the practice a bad name	0/0/0/1/0	1/0
	Demographical characteristics of the patient	Patients’ demographical characteristics influencing doctors’ and nurses’ screening and advice performance	0/1/0/0/0	1/0
	SBI delivery impedes caring for the patient	Doctors’ and nurses’belief that bringing alcohol into the discussion impedes the comprehensive care of the patient	1/0/0/0/0	1/0
	Uncomfortable task	Doctors’ and nurses’ expectation of feeling unease or awkward when conducting alcohol screening and brief interventions	0/0/1/0/1	1/1
	Patients with alcohol problems do not attend their appointments	Doctors’ and nurses’expectation that patients with alcohol problems would not attend appointments to address their drinking	0/1/0/0/0	1/0
Motivation—social/professional role and identity	Role legitimacy	The extent to which doctors and nursesbelieve they have a legitimate role in addressing alcohol issues in their patients	15/4/4/1/2	41/0
	Professional responsibility	The extent to which doctors and nurses find addressing alcohol in their patients to be their responsibility	12/2/4/1/0	24/0
	Disease model training	An approach to the patient in that health providers ask about alcohol only when the patient present with specific symptoms and/or signs	7/2/4/1/0	14/0
	Doctors and nurses own drinking habits	The use of doctors and nurses own drinking behaviour as a benchmark to define whether or not a patient drinks excessively	4/2/3/0/0	9/0
	Doctors’ and nurses’ permissiveness towards alcohol	Doctors’ and nurses’ tolerance or acceptability towards their patients’ alcohol consumption	3/3/0/0/0	7/0
	Role security	The extent to which doctors and nurses feel secure in their role when addressing alcohol issues in their patients	3/0/0/0/2	5/0
	Doctors’ and nurses’ attitudes towards discussing alcohol with patients	The way doctors and nurses feel or think about asking and advising their patients about their drinking	1/0/1/0/1	3/0
	Patients’ feelings when asked about their drinking	Doctors’ and nurses’ beliefs about how patients would feel if asked and advised about alcohol	0/2/1/1/0	3/3
	Demographical characteristics of the PHC professionals	Doctors’ and nurses’ demographical characteristics influencing thier screening and advice performance	0/0/1/0/0	1/0
	Demographical characteristics of the patient	Patients’ demographical characteristics influencing doctors’ and nurses’ screening and advice performance	0/1/0/0/0	1/0
	Therapeutic relation with the patient	The therapeutic alliance that is established between a healthcare professional and a patient	1/0/2/1/1	1/4
	Feedback on the results of delivering SBI	Information about doctors’ and nurses’ performance concerning screening, advice and/or effectiveness of their actions	0/1/1/0/1	1/2
Motivation—emotion	Satisfaction when working with at-risk drinkers	The extent to which doctors and nurses feel rewarded when working with at-risk drinkers	13/0/1/0/0	19/0
	Uncomfortable task	Doctors’ and nurses’ expectation of feeling unease or awkward when conducting alcohol screening and brief interventions	5/6/3/0/3	16/1
	Patients’ feelings when asked about their drinking	Doctors’ and nurses’ beliefs about how patients would feel if asked and advised about alcohol	0/2/5/1/0	7/3
	Frustrating task	Doctors’ and nurses’ beliefs about how they would feel if they were to implement alcohol screening and brief interventions	2/2/0/1/1	5/1
	Therapeutic commitment	Doctors’ and nurses’ predisposition to working therapeutically with people who have excessive alcohol consumption	3/0/0/0/2	5/0
	Self-esteem when working with at-risk drinkers	Doctors’ and nurses’ perceived self-worth when working with at-risk drinkers	1/0/1/1/1	2/3
	Doctors and nurses own drinking habits	The use of doctors and nurses own drinking behaviour as a benchmark to define whether or not a patient drinks excessively	0/2/0/0/0	3/0
	Motivation to work with at-risk drinkers	The extent to which doctors and nurses want to work with at-risk drinkers	4/2/1/0/1	1/16
Motivation—intentions	Motivation to work with at-risk drinkers	The extent to which doctors and nurses want to work with at-risk drinkers	15/2/2/2/1	18/16
	Therapeutic commitment	Doctors’ and nurses’ predisposition to working therapeutically with people who have excessive alcohol consumption	3/0/0/0/2	5/0
Motivation—reinforcement	Incentives	Doctors’ and nurses’ beliefs about what they would gain by implementing alcohol screening and brief interventions	7/1/4/1/2	13/22
Motivation—optimism	Beliefs about the ability to deliver SBI and in helping patients to cut down	Doctors’ and nurses’ beliefs about, and/or confidence in, the effectiveness of their skills to screen and advise patients to reduce their alcohol intake	3/2/1/1/1	4/6
Motivation—goals	Importance / Priority given to alcohol issues	Importance / priority given to alcohol issues by doctors and nurses when compared to other risk factors or tasks	5/5/3/0/0	13/1
	Time	Time-related factors doctors and nurses believe to affect their capability to implement alcohol screening and brief interventions	5/4/3/0/2	7/14
Opportunity—environmental context and resources	Time	Time-related factors doctors and nurses believe to affect their capability to implement alcohol screening and brief interventions	16/7/11/3/5	45/14
	Support	The extent to which doctors and nurses feel to be working in supporting environment to address alcohol problems	24/3/5/2/4	30/57
	Resources	The availability of materials, tools or any other thing that doctors and nurses feel they need to screen and advise at-risk drinkers	9/3/5/1/3	22/21
	Patients’ denial of the problem and resistance to accepting treatment	The extent to which doctors and nurses agree patient denial of the problem and resistance to treatment influence their decision to deliver screening and brief intervention	6/3/1/1/0	14/0
	Patients’ feelings when asked about their drinking	Doctors’ and nurses’ beliefs about how patients would feel if asked and advised about alcohol	5/3/3/1/0	12/3
	Organization for preventive counselling	Doctors’ and nurses’ perception of the presence or absence of organization/systematic strategies to implement alcohol screening and brief advice	6/4/7/0/3	9/19
	Patients’ beliefs about alcohol	Doctors’ and nurses’ perceptions of the conceptions patients have about the effects of alcohol, either beneficial or detrimental	1/3/1/0/0	8/1
	Incentives for patients	Something (e.g. reimbursement) doctors and nurses think would encourage patients to seek alcohol counselling	5/1/1/0/0	7/0
	Patients with alcohol problems do not attend their appointments	Doctors’ and nurses’ perception that at-risk drinkers are not interested and frequently miss follow-up consultations	0/2/0/1/1	4/0
	Patients’ receptiveness to alcohol interventions	The extent to which doctors and nurses think patients are open to be asked and advised about their drinking	0/2/1/1/2	4/4
	Delivering SBI can make other patients suffer	Doctors’ and nurses’ belief that implementing alcohol screening and brief interventions could harm other patients	1/0/0/1/0	2/0
	Familiarity with the patient	The level of acquaintance between the primary health care provider and the patient	0/1/1/0/0	1/1
Opportunity—social influences	Patients’ feelings when asked about their drinking	Doctors’ and nurses’ beliefs about how patients would feel if asked and advised about alcohol	5/4/5/1/0	17/3
	Patients’ reactions when asked about alcohol	Doctors’ and nurses’ beliefs about how patients would react if asked and advised about alcohol	3/5/1/2/1	10/3
	Doctors’ and nurses’ permissiveness towards alcohol	Doctors’ and nurses’ tolerance or acceptability towards their patients’ alcohol consumption	3/3/0/0/0	7/0
	Patients seeking help	Patients asking primary care doctors or nurses for help or advice about their drinking by their own initiative	4/4/0/0/0	4/6
	Support	The extent to which doctors and nurses feel to be working in supporting environment to address alcohol problems	11/3/5/2/2	3/57
	Patients’ receptiveness to alcohol interventions	The extent to which doctors and nurses think patients are open to be asked and advised about their drinking	0/2/1/1/2	4/4
	Role legitimacy	The extent to which doctors and nurses believe they have a legitimate role in addressing alcohol issues in their patients	0/1/1/0/0	2/0
	Presence of third parties in the consultation	Having relatives, friends or other persons attending the consultation with the patient	0/1/0/0/0	1/0

The most commonly reported barrier-related themes were related to ‘beliefs about their ability to deliver SBI and to help patients to cut down’ (*n* = 62 data units), ‘alcohol-related knowledge’ (*n* = 58 data units), and ‘time’ (*n* = 50 data units). A total of 253 data items pertaining to facilitators were extracted. All facilitator items related to or addressed one of the 46 barrier themes. Together, the facilitator items mapped onto 22 of the 46 themes. The most commonly reported facilitator-related themes were related to ‘support’ (*n* = 57 data units), ‘training’ (*n* = 25 data units) and ‘difficult task’ (*n* = 24 data units).

TDF domains are numbered as originally designated [30]. All 46 identified themes are mapped to at least one of the three components of the COM-B system and to at least one of the 14 domains of the TDF (Table 2). Additional files 5 and 6 provide a complete description of the barriers and facilitators extracted.

### Capability (COM-B component 1)

Thirteen themes relate to the capability component of the COM-B system, which includes four TDF domains (Knowledge; Skills; Memory, Attention and Decision Processes; Behavioural regulation). These 13 themes emerged from 68 studies from 26 countries (Table 3). Most studies (*n* = 40) were quantitative in design and reported data mainly from GPs (*n* = 49).

**Table 3:** Themes coded to each of the TDF domains within the capability component of the COM-B system

TDF	Theme	Countries	References on barriers	References on facilitators
Knowledge	Alcohol-related knowledge	UK(8); Finland(6); Sweden(4); Multicountry(3); Norway(3); Australia(2); New Zealand(2); South Africa(2); Spain(2); USA(2); Brazil(1); Canada(1); France(1); Netherlands(1); Portugal(1); Slovenia(1); Sri Lanka(1)	[16, 20, 23, 36–38, 41, 42, 47, 52, 56, 57, 62, 64–67, 70, 73–75, 78, 83, 84, 87, 89, 92, 94, 96, 97, 99, 101, 103, 105, 108, 111, 114]	[39, 55, 70, 73, 79, 100]
	Disease model training	Finland(3); Sweden(1); UK(1)	[38, 41, 42, 61, 74]	
	Doctors and nurses own drinking habits	UK(2); Norway(1)	[77, 87, 89]	
	Alcohol being perceived as having health benefits	Finland(1); Sweden(1); UK(1)	[20, 38, 42]	
	Patients’ receptiveness to alcohol interventions	Australia(1); Finland(1); New Zealand(1); Norway(1); UK(1); USA(1)	[42, 65, 105]	[58, 87, 97]
	Knowledge of support services	Sweden(1); UK(1)	[70, 101]	
Skills	Training	UK(13); Sweden(5); Multicountry(4); USA(3); Canada(2); Finland(2); Spain(2); Australia(1); Brazil(1); Denmark(1); Italy(1); Netherlands(1); New Zealand(1); Portugal(1); Slovenia(1); South Africa(1); Sri Lanka(1)	[16, 20, 23, 36, 41, 44, 46, 47, 49, 52, 57, 59–65, 67, 68, 70, 76, 78, 79, 83, 89, 90, 92, 94–96, 99, 110, 111]	[23, 36, 39, 48, 52, 55, 72, 73, 78–80, 84, 92, 101]
	Role adequacy	UK(9); Australia(3); USA(3); Multicountry(2); Sweden(2); Canada(1); Denmark(1); Finland(1); New Zealand(1); Norway(1); Portugal(1); Slovenia(1); South Africa(1); Spain(1); Sri Lanka(1)	[16, 20, 23, 36, 44, 47, 49, 52, 57, 59, 62, 64, 65, 67, 70, 78, 83, 84, 87, 92, 95, 97, 103–106, 108, 111, 116]	
	Demographical characteristics of the PHC professionals	Germany(1); Norway(1)	[51, 100]	
Memory, attention and decision processes	Demographical characteristics of the patient	Finland(1); Germany(1); Sweden(1); UK(1); USA(1)	[41, 51, 74, 105, 115]	
	Remembering	Finland(1); Sweden(1)	[41, 74]	
	Feedback on the results of delivering SBI	UK(2); Finland(1)	[42]	[71, 109]
Behaviour regulation	Organization for preventive counselling	Sweden(4); UK(4); Slovenia(2); Canada(1); Finland(1); Multicountry(1); Netherlands(1); New Zealand(1); Norway(1); South Africa(1); USA(1)	[47, 74, 78, 83, 103, 109]	[39, 41, 54, 70, 71, 73, 76, 80, 91, 93, 97, 100]

#### Skills—TDF domain no. 2

##### Theme: Training

In general, both GPs and nurses reported a lack of training in dealing with alcohol problems. The majority of the GPs thought their medical training was inadequate to address alcohol issues in their patients. Three survey studies from the UK found that only a minority of the GPs and nurses received alcohol-specific training since graduation [59–61]. In 9 survey studies, the majority of the GPs and nurses who received training reported that those programmes lasted less than four hours [20, 23, 44, 46, 47, 70, 76, 78, 79]. Several studies on both GPs and nurses reported availability of educational and training programmes as an important facilitator [23, 36, 55, 73, 78, 84].

##### Theme: Role adequacy

Mixed evidence was found concerning GPs’ and nurses’ appraisal of their skills in detecting and advising at-risk drinkers. On the one hand, the majority of the GPs in 9 [20, 44, 47, 57, 59, 62, 67, 83, 104], and of the nurses in 3 quantitative studies [20, 59, 84] felt they were not skilled enough to deliver alcohol SBI; on the other hand, the majority of the GPs in 14 [16, 20, 23, 36, 52, 62, 64, 70, 78, 83, 95, 106, 108, 111], and of the nurses in 2 quantitative studies [36, 70] reported the opposite.

#### Knowledge—TDF domain no. 1

##### Theme: Alcohol-related knowledge

A total of 53 data units from 35 studies reporting on barriers were extracted. Most data came from GPs (*n* = 34). Alcohol-related knowledge included issues of self-reported knowledge of alcohol SBI concepts (e.g. the definition of sensible drinking limits, the content of a brief intervention), and familiarity with guidelines and screening tools. One Spanish study found that 60% of the GPs had not received alcohol-specific education during medical school [94]. A varying degree of both GPs and nurses in 2 survey studies indicated alcohol-specific education as a facilitator [70, 73].

##### Theme: Disease model training

Four studies (3 qualitative and 1 quantitative) from the Nordic countries mentioned that GPs asked their patients about alcohol only if there was something that made them suspect the patient was a heavy drinker [38, 41, 42, 74]. Notwithstanding, a quantitative study from the UK reported that only 4% of the GPs agreed that their role was to treat alcohol-related medical complications only [61].

Four other less frequently mentioned themes were linked to this TDF domain (Table 3).

#### Memory, attention and decision processes—TDF domain no. 10

##### Theme: Demographical characteristics of the patient

Six pieces of data from 3 qualitative, 1 quantitative and 1 mixed methods studies alluded that both GPs and nurses screening activities were influenced by patients’ characteristics [41, 51, 74, 105, 115]. Older patients and being a female were found to be at lower odds of being detected as problem drinkers, whilst visiting the GP more than 5 times within the last year increased the chances of detection.

##### Theme: Remembering

Asking patients about alcohol was found easy to forget in 2 qualitative studies on GPs from the Nordic countries [41, 74].

##### Theme: Feedback on the results of delivering SBI

One interview study from Finland found that GPs were unaware of whether or not patients they advised reduced their drinking because GPs do not schedule follow-up appointments [42].

#### Behavioural regulation—TDF domain no. 14

##### Theme: Organization for preventive counselling

Three survey studies showed that 40 to 86% of the GPs believed general practices are not organized to do preventive counselling [47, 78, 83]. GPs in 3 qualitative studies mentioned that implementation strategies for routine screening of at-risk drinkers were lacking [74, 103, 109]. GPs and nurses often cited improving professional teamwork (e.g. having a practice nurse delivering SBI, having receptionists giving patients screening tools) as a facilitator [39, 41, 54, 70, 71, 73, 76, 80, 91, 93, 97, 99].

### Motivation (COM-B component 2)

The 33 themes in the motivation component of the COM-B system, which includes eight TDF domains (Social/professional role and identity; Beliefs about capabilities; Optimism; Beliefs about Consequences; Reinforcement; Intentions; Goals; Emotion), emerged from 75 studies from 30 countries (Table 4). The majority of the studies (*n* = 43) were quantitative in design and reported data mainly from GPs (*n* = 54).

**Table 4:** Themes coded to each of the TDF domains within the motivation component of the COM-B system

TDF	Theme	Countries	References on barriers	References on facilitators
Beliefs about capabilities	Beliefs about the ability to deliver SBI and in helping patients to cut down	UK(11); Australia(5); Multicountry(5); Finland(3); Sweden(3); USA(3); Canada(2); Denmark(1); New Zealand(2); Netherlands(1); South Africa(1); Spain(1); Sri Lanka(1)	[20, 36, 37, 40, 41, 44–48, 50, 52, 53, 55, 59–62, 64, 65, 67, 72, 75, 78, 81, 84, 85, 97, 101, 104–109, 111, 116]	[48, 49, 71, 78, 100]
	Time	Sweden(7); Australia(5); UK(5); USA(4); Finland(2); Norway(2); Canada(1); Denmark(1); Multicountry(1); Netherlands(1); New Zealand(1); Portugal(1); Slovenia(1); Sri Lanka(1)	[16, 20, 37, 41, 48, 49, 52, 54, 58, 62, 63, 65, 67, 70, 72–74, 87, 91, 92, 95, 97, 99, 105–107, 109, 111]	[39, 43, 70, 71, 80, 92, 93, 97]
	Difficult task	UK(8); Australia(3); Canada(3); Finland(3); Norway(3); Multicountry(2); Sweden(2); Brazil(1); Denmark(1); Netherlands(1); New Zealand(1); Portugal(1); South Africa(1); Sri Lanka(1)	[16, 23, 37, 41, 42, 49, 54, 59–62, 67, 68, 73, 78, 83, 86, 90, 92, 97, 99, 102, 105, 107, 109, 113]	[39, 41, 58, 62, 71, 86, 87, 97, 99, 105, 109]
	Therapeutic commitment	Multicountry(4); Netherlands(1)	[45–47, 50, 81]	
	Self-esteem when working with at-risk drinkers	UK(3); Portugal(1); Sweden(1)	[16, 20, 23, 78]	[71]
	Disease model training	Finland(1)	[41]	
	Patients’ misbeliefs about alcohol	UK(1)	[105]	
	Demographical characteristics of the patient	New Zealand(1)	[97]	
Beliefs about consequences	Effectiveness of SBI	UK(6); Finland(3); Sweden(3); Australia(2); Multicountry(2); Norway(2); Canada(1); Denmark(1); Italy(1); Netherlands(1); New Zealand(1); South Africa(1); Spain(1)	[20, 23, 36, 38, 42, 45, 49, 55, 57, 64, 70, 73, 78, 83, 84, 86, 91, 106, 107, 110]	[23, 36, 39, 47, 71, 78, 84, 87, 92, 109]
	Patients’ feelings when asked about their drinking	Norway(3); UK(3); Finland(2); Multicountry(2); USA(2); Australia(1); Brazil(1); France(1); New Zealand(1); Slovenia(1); Sweden(1)	[20, 23, 41, 42, 47, 56, 71, 78, 84, 87, 92, 93, 97, 99, 103, 113, 115]	[58, 93]
	Therapeutic relation with the patient	Sweden(3); UK(2); Canada(1); Denmark(1); Finland(1); France(1); New Zealand(1); Norway(1); Slovenia(1); USA(1)	[37, 48, 49, 56, 72, 74, 91, 97, 99, 103, 109, 115]	[49, 74, 84, 99]
	Reliability of the answers of the patients when asked about alcohol	Finland(2); Denmark(1); Multicountry (1); New Zealand(1); Norway(1); Sri Lanka(1); Sweden(1); UK(1)	[37, 42, 48, 49, 67, 89, 92, 97, 99]	
	Patients’ reactions when asked about alcohol	Sweden(3); UK(3); Australia(1); Denmark(1); Finland(1); Multicountry(1)	[70, 73, 84, 89, 92, 95, 102]	[41, 48, 49]
	Patients’ receptiveness to alcohol interventions	Finland(2); USA(2); Australia(1); New Zealand(1); Norway(1); Slovenia(1); Sweden(1); UK(1)	[36, 42, 63, 65, 73, 103, 105]	[58, 87, 97]
	Frustrating task	UK(3); Canada(1); Portugal(1); Sweden(1)	[16, 57, 62, 77, 109]	[48]
	Alcohol being perceived as having health benefits	Finland(1); Sweden(1); UK(1)	[20, 42, 78]	
	Incentives	UK(3); Australia(1); Finland(1); Multicountry(1); Netherlands(1); Slovenia(1); Sweden(1)	[95, 107]	[20, 23, 36, 39, 78, 80, 92]
	Time	Sweden(2); Australia(1); Finland(1); Multicountry(1); Netherlands(1); New Zealand(1); Slovenia(1); UK(1); USA(1)	[37, 48]	[39, 43, 70, 71, 80, 92, 93, 97]
	Delivering SBI can make other patients suffer	Sweden(1); UK(1)	[73, 95]	
	Bad publicity	UK(1)	[95]	
	Demographical characteristics of the patient	UK(1)	[89]	
	SBI delivery impedes caring for other patients	Finland(1)	[38]	
	Uncomfortable task	Australia(1); Netherlands(1)	[107]	[39]
	Patients with alcohol problems do not attend their appointments	New Zealand(1)	[97]	
Social/professional role and identity	Role legitimacy	UK(7); Finland(5); Sweden(3); Canada(2); New Zealand(2); Australia(1); Denmark(1); Norway(1); Portugal(1); Slovenia(1); South Africa(1); Spain(1)	[16, 23, 36–38, 44, 49, 52, 55, 57, 62, 64, 72–75, 78, 83, 89, 97, 99, 102, 105, 108, 109]	
	Professional responsibility	UK(7); Sweden(3); Finland(2); New Zealand(2); Australia(1); Multicountry(1); South Africa(1); Sri Lanka(1); USA(1)	[23, 37, 38, 47, 55, 59–62, 65, 67, 72–74, 78, 83, 97, 105, 107]	
	Disease model training	UK(4); Sweden(3); Finland(2); Australia(1); Multicountry(1); Norway(1); South Africa(1); Sri Lanka(1)	[23, 41, 42, 47, 58, 61, 67, 72–74, 78, 83, 88, 99]	
	Doctors and nurses own drinking habits	UK(4); Canada(1); Multicountry(1); Norway(1); Slovenia(1); Sweden(1)	[23, 47, 74, 77, 78, 87, 89, 98, 103]	
	Doctors’ and nurses’ permissiveness towards alcohol	UK(2); Finland(1); Multicountry(1); Sweden(1); USA(1)	[20, 23, 38, 47, 89, 115]	
	Role security	Multicountry(4); Netherlands(1)	[45–47, 50, 81]	
	Doctors’ and nurses’ attitudes towards discussing alcohol with patients	Finland(2); Denmark(1)	[36, 37, 49]	
	Patients’ feelings when asked about their drinking	Finland(2); Australia(1); USA(1)	[41, 42, 93]	
	Demographical characteristics of the PHC professionals	Australia(1); Canada(1)	[107, 109]	
	Demographical characteristics of the patient	UK(1)	[89]	
	Therapeutic relation with the patient	Denmark(1); Finland(1); Norway(1); Sweden(1); UK(1)	[38]	[49, 74, 84, 99]
	Feedback on the results of delivering SBI	UK(2); Finland(1)	[42]	[71, 109]
Emotion	Uncomfortable task	UK(5); Finland(2); USA(2); Canada(1); France(1); Multicountry(1); Netherlands(1); New Zealand(1); Norway(1); South Africa(1); Sweden(1)	[23, 37, 41, 47, 54, 56, 77, 78, 83, 89, 91, 97, 99, 109, 115, 116]	[39]
	Satisfaction when working with at-risk drinkers	UK(8); Sweden(2); Canada(1); Portugal(1); Spain(1); Sri Lanka(1)	[16, 20, 23, 44, 57, 59–62, 67, 73, 78, 94, 108]	
	Patients’ feelings when asked about their drinking	Norway(3); USA(2); Australia(1); New Zealand(1); UK(1);	[71, 86, 87, 97, 99, 115]	[58, 93]
	Frustrating task	UK(3); Canada(1); Portugal(1); Sweden(1)	[16, 57, 62, 77, 109]	[48]
	Therapeutic commitment	Multicountry(4); Netherlands(1)	[45–47, 50, 81]	
	Self-esteem when working with at-risk drinkers	UK(2); Canada(1)	[62, 108]	[71]
	Doctors and nurses own drinking habits	UK(2)	[77, 89]	
	Motivation to work with at-risk drinkers	UK(3); Multicountry(1); Netherlands(1); Norway(1); Sri Lanka(1); Sweden(1)	[105]	[39, 47, 48, 59, 60, 67, 99]
Intentions	Motivation to work with at-risk drinkers	UK(9); Sweden(3); Australia(2); Spain(2); Canada(1); Multicountry(1); Netherlands(1); Portugal(1); Sri Lanka(1); USA(1)	[16, 20, 23, 44, 57, 62, 64–66, 78, 94, 95, 105, 107, 108]	[39, 47, 48, 59, 60, 67, 99]
	Therapeutic commitment	Multicountry(4); Netherlands(1)	[45–47, 50, 81]	
Reinforcement	Incentives for delivering SBI	UK(3); Australia(2); Multicountry(2); Slovenia(2); Finland(1); Netherlands(1); Norway(1); Portugal(1); South Africa(1); Sweden(1)	[16, 23, 47, 78, 83, 92, 95, 99, 103, 106, 107]	[20, 23, 36, 39, 78, 80, 92]
Goals	Importance/priority given to alcohol issues	UK(4); Sweden(3); Multicountry(2); Norway(2); Finland(1); USA(1)	[20, 23, 41, 47, 48, 65, 71, 74, 78, 88, 92, 105]	[99]
	Time	UK(4); Australia(2); Multicountry(2); Netherlands(1); New Zealand(1); Slovenia(1); South Africa(1); Sweden(1); USA(1)	[23, 47, 78, 83, 92, 105, 106]	[39, 43, 70, 71, 80, 92, 93, 97]
Optimism	Beliefs about the ability to deliver SBI and in helping patients to cut down	UK(3); Denmark(1), Multicountry(1); New Zealand(1); Norway(1); Sweden(1)	[55, 62, 92]	[48, 49, 71, 78, 100]

#### Beliefs about capabilities—TDF domain no. 4

##### Theme: Beliefs about the ability to deliver SBI and in helping patients to cut down

Twenty-three studies reported on how GPs felt about their abilities for screening and advising at-risk drinkers, of which 16 found a majority of GPs believed they were confident in their abilities [20, 36, 40, 44–47, 50, 52, 59–62, 64, 65, 67, 78, 81, 83, 104, 106, 108, 111] compared with 1 of 3 studies involving nurses [20, 36, 59]. Notwithstanding, the majority of the GPs in 7 from a total of 11 studies [20, 53, 55, 59–62, 65, 78, 83, 108], and of the nurses in 2 studies [20, 85], did not feel their advice would have much impact. GPs and nurses reported more training for improving counselling skills [20, 49, 78, 83] and feedback on successful cases [71] as facilitators.

##### Theme: Time

Lack of time was cited as a barrier, mainly by GPs, in 28 studies. Two main sub-themes were identified: having competing demands (e.g. needing to attend patients with multiple health problems); and thinking that alcohol SBI is too time consuming. More time per consultation, more experience in delivering brief interventions and simplifying the screening process (e.g. short and simple screening tools, giving patients self-report questionnaires) are examples of reported facilitators [39, 43, 93, 97].

Six other less frequently mentioned themes were linked to this TDF domain (Table 4).

#### Beliefs about Consequences—TDF domain no. 6

##### Theme: Effectiveness of SBI

Mixed evidence was found concerning whether or not GPs believed in the effectiveness of brief interventions for reducing alcohol consumption. In 4 quantitative and 4 qualitative studies GPs were sceptical that patients would follow their advice [20, 42, 47, 49, 78, 83, 91, 107]; data from 6 quantitative and 1 qualitative studies point otherwise [23, 64, 70, 73, 86, 106, 110]. Three studies on nurses found that most believed in the efficacy of brief interventions [70, 73, 84]. More information about the effectiveness of brief interventions [23, 36, 39, 47, 78, 84, 87, 92] and feedback on successful cases [71, 109] were identified as implementation facilitators.

##### Theme: Patients’ feelings when asked about their drinking

Evidence from several qualitative studies suggest that GPs and nurses might be afraid to offend their patients by asking them about alcohol. This issue was addressed in 5 survey studies among GPs, of which 4 found a majority of GPs did not believe patients would resent being asked [20, 23, 47, 56, 78]. Increasing experience with screening and normalizing alcohol questions were reported as facilitators [93].

Fourteen other less frequently mentioned themes were linked to this TDF domain (Table 4).

#### Social/professional role and identity—TDF domain no. 3

##### Theme: Role legitimacy

In general, the majority of both GPs and nurses agreed that identifying and providing alcohol-related advice is a natural part of their job. Nearly all GPs in 3 studies from the UK and Canada believed they have the right to ask patients about alcohol and that their patients share this view [57, 62, 108].

##### Theme: Professional responsibility

Believing that preventing alcohol problems is a GP responsibility was found to vary substantially from country to country. On the one hand, the majority of the GPs in 1 multicountry and 1 South African studies reported that these problems were not their responsibility [47, 67]; on the other hand, the majority of the GPs in 2 studies from the UK and 1 study from the USA thought the opposite [23, 65, 78].

##### Theme: Disease model training

A varying number of GPs agreed to have disease model training and that they do not think about prevention. Data, mainly from qualitative studies, suggested that GPs and nurses do not screen systematically for alcohol but only when they suspected heavy consumption, or when the patient’s complaint was likely to be alcohol-related [41, 42, 58, 67, 72–74, 88].

Nine other less frequently mentioned themes were linked to this TDF domain (Table 4).

#### Emotion—TDF domain no. 13

##### Theme: Uncomfortable task

Several GPs and nurses expressed feeling uneasy when asking patients about their drinking. In 10 qualitative studies, primary health care (PHC) providers considered asking about alcohol a delicate task because alcohol is viewed as a sensitive issue, making them feel uncomfortable [37, 41, 54, 77, 89, 91, 97, 99, 109, 115]. Notwithstanding, the majority of the GPs in 4 from a total of 6 quantitative studies reported feeling comfortable asking about alcohol [23, 47, 56, 78, 83, 116]. Destigmatizing problematic alcohol use was identified as a facilitator in 1 qualitative study [39].

##### Theme: Satisfaction when working with at-risk drinkers

With the notable exception of 1 study on GPs from Sri Lanka [67], the majority of the GPs in the remaining 13 studies addressing this issue (8 from the UK), and of the nurses in 3 studies (2 from Sweden and 1 from the UK) reported feeling unsatisfied advising patients to cut down [16, 20, 23, 44, 57, 59–62, 73, 78, 94, 108].

Six other less frequently mentioned themes were linked to this TDF domain (Table 4).

#### Intentions—TDF domain no. 8

##### Theme: Motivation to work with at-risk drinkers

In 2 qualitative studies from Australia and the UK, GPs acknowledged they were not interested in dealing with alcohol problems [105, 107]. The majority of the GPs in 8 from a total of 10 quantitative studies felt unmotivated to work with at-risk drinkers [16, 23, 44, 57, 62, 66, 78, 94, 95, 108]. In 1 survey study from Sweden, nurses scored neutral on a motivational scale from 1 to 7 [20]. The majority of the GPs from several countries [47, 59, 60, 67], and of the nurses in 1 UK-based study [59], reported that more training in brief interventions would increase their motivation to work with at-risk drinkers. Seventeen to 33% of the GPs from Sri Lanka and the UK agreed they would be more willing to work with at-risk drinkers if financial incentives were provided [59, 67].

##### Theme: Therapeutic commitment

Five quantitative studies (4 on GPs and 1 in both GPs and nurses) employed a validated scale for measuring GPs’ and nurses’ predisposition for working therapeutically with at-risk drinkers [45–47, 50, 81]. All 5 studies reported that the majority of these professionals were not therapeutically committed.

#### Goals—TDF domain no. 9

##### Theme: Importance/priority given to alcohol issues

Fourteen to 54% of the GPs in 3 quantitative studies considered alcohol an unimportant issue in PHC [23, 47, 78]. Creating a specific billing code for this area was reported by some Norwegian GPs as a facilitator to increase GPs awareness of the importance of alcohol-related problems [99].

##### Theme: Time

Alcohol was not a goal priority for GPs because they were too busy, which makes them neglect alcohol issues in favour of other presenting problems [23, 47, 78, 83, 92, 105, 106]. Implementing a short questionnaire in the registration system [39] and increasing knowledge that a brief intervention costs little time and can be effective [39] were suggested as facilitators.

#### Reinforcement—TDF domain no. 7

##### Theme: Incentives

The majority of the GPs in 3 quantitative studies reported that alcohol SBI activities were not reimbursable under government health schemes [47, 78, 83]. Three qualitative studies reported that GPs and nurses would feel incentivized if financial reimbursement for providing alcohol brief interventions was available [39, 80, 105]; however, only 24% of the GPs and nurses in 2 survey studies from the Nordic countries agreed with this [20, 36].

#### Optimism—TDF domain no. 5

##### Theme: Beliefs about the ability to deliver SBI and in helping patients to cut down

Two quantitative studies from New Zealand and the UK found that 13 to 28% of the GPs felt pessimistic about what they could do to help at-risk drinkers [55, 62]. More training for improving counselling skills [49, 78] and feedback on successful cases [71] were reported as facilitators.

### Opportunity (COM-B component 3)

The 17 themes in the opportunity component of the COM-B system, which includes two TDF domains (Environmental context and resources; Social influences), emerged from 66 studies from 25 countries (Table 5). The majority of the studies (*n* = 33) were quantitative in design and reported data mainly from GPs alone (*n* = 44).

**Table 5:** Themes coded to each of the TDF domains within the opportunity component of the COM-B system

TDF	Theme	Countries	References on barriers	References on facilitators
Environmental context and resources	Time	UK(10); Sweden(7); Australia(5); USA(5); Finland(2); Multicountry(2); Norway(2); Slovenia(2); Canada(1); Denmark(1); Netherlands(1); New Zealand(1); Portugal(1); South Africa(1); Sri Lanka(1)	[16, 20, 23, 37, 41, 47–49, 52, 54, 58–62, 65, 67, 70–74, 78, 83, 87, 91–93, 95, 97, 99, 103, 105–107, 109, 111, 116]	[39, 43, 70, 71, 80, 92, 93, 97]
	Support	UK(12); Multicountry(4); Canada(3); Finland(3); New Zealand(2); Norway(2); South Africa(2); Sweden(2); USA(2); Brazil(1); France(1); Italy(1); Netherlands(1); Slovenia(1); Sri Lanka(1);	[46, 47, 55–57, 61, 63, 65, 70, 71, 75, 78, 82, 83, 88, 92, 96, 101, 105, 108, 109, 112, 114]	[20, 23, 36, 37, 39, 47, 59, 60, 62, 67, 70, 71, 78, 81, 84, 92, 97, 99, 100, 105, 110]
	Resources	Finland(4); Sweden(3); UK(3); Australia(2); Multicountry(2); Canada(1); Netherlands(1); New Zealand(1); Norway(1); Slovenia(1); South Africa(1); USA(1)	[20, 23, 38, 41, 42, 47, 58, 63, 69, 74, 78, 83, 103, 109]	[20, 23, 37, 39, 42, 47, 55, 70, 78, 88, 92, 99]
	Patients’ denial of the problem and resistance to accepting treatment	Australia(2); USA(2); Brazil(1); Canada(1); France(1); Finland(1); New Zealand(1); Norway(1); Sweden(1)	[41, 52, 56, 58, 63, 65, 73, 86, 97, 112, 113]	
	Patients’ feelings when asked about their drinking	UK(3); Multicountry(2); USA(2); Australia(1); France(1); New Zealand(1); Slovenia(1); Sweden(1)	[20, 23, 47, 56, 71, 78, 92, 97, 103, 115]	[58, 93]
	Organization for preventive counselling	UK(5); Sweden(4); Slovenia(2); Australia(1); Canada(1); Finland(1); Multicountry(1); Netherlands(1); New Zealand(1); Norway(1); South Africa(1); USA(1)	[47, 52, 74, 78, 83, 89, 103, 109]	[39, 41, 54, 70, 71, 73, 76, 80, 91, 93, 97, 99]
	Incentives for patients	Multicountry(2); UK(2); Canada(1); Italy(1); South Africa(1)	[47, 78, 83, 88, 92, 110, 112]	
	Patients’ beliefs about alcohol	Finland(2); UK(2); New Zealand(1)	[37, 38, 89, 105]	[97]
	Patients with alcohol problems do not attend their appointments	UK(2); Denmark(1); New Zealand(1)	[49, 95, 97, 105]	
	Patients’ receptiveness to alcohol interventions	Australia(1); Denmark(1); New Zealand(1); Norway(1); UK(1); USA(1)	[49, 105, 116]	[58, 87, 97]
	Delivering SBI can make other patients suffer	Sweden(1); UK(1)	[73, 95]	
	Familiarity with the patient	UK(2)	[105]	[71]
Social support	Patients’ feelings when asked about their drinking	UK(4); Multicountry(2); USA(2); Australia(1); Brazil(1); France(1); New Zealand(1); Norway(1); Slovenia(1); Sweden(1)	[20, 23, 47, 56, 71, 78, 88, 92, 93, 97, 99, 103, 113, 115]	[58, 93]
	Patients’ reactions when asked about alcohol	UK(4); Sweden(3); Australia(1); Denmark(1); Finland(1); Multicountry(1); Norway(1)	[49, 70, 73, 84, 86, 89, 92, 95, 102, 105]	[41, 48, 49]
	Doctors’ and nurses’ permissiveness towards alcohol	UK(2); Finland(1); Multicountry(1); Sweden(1); USA(1)	[20, 23, 41, 47, 89, 115]	
	Patients seeking help	Finland(2); Multicountry(2); UK(2); Brazil(1); France(1)	[41, 42, 56, 113]	[23, 41, 47, 78, 92]
	Support	UK(8); Multicountry(3); Finland(2); Norway(2); Slovenia(2); Sweden(2); Italy(1); Netherlands(1); New Zealand(1); Sri Lanka(1)	[82, 99, 103]	[20, 23, 36, 37, 39, 47, 59, 62, 67, 70, 71, 78, 80, 84, 92, 97, 99, 100, 105, 110]
	Patients’ receptiveness to alcohol interventions	Australia(1); Denmark(1); New Zealand(1); Norway(1); UK(1); USA(1)	[49, 105, 116]	[58, 87, 97]
	Role legitimacy	Norway(1); USA(1)	[99, 115]	
	Presence of third parties in the consultation	New Zealand(1)	[97]	

#### Environmental context and resources—TDF domain no. 11

##### Theme: Time

GPs and nurses often cited time constraints as a barrier for implementing alcohol SBI. For some doctors and nurses, alcohol SBI was too time-consuming [72, 95, 106] and they were already too busy dealing with other problems [23, 47, 78, 83, 92, 106]. More time per consultation [39, 70, 80, 92, 97], more experience in delivering brief interventions [93], and simpler screening processes (e.g. short and simple screening tools, giving patients self-report questionnaires) [39] were reported as facilitators.

##### Theme: Support

Data from both qualitative and quantitative studies show that, in general, providers felt they could be working in a more supportive environment for delivering alcohol SBI. The majority of the GPs in 3 survey studies reported lack of support from government health policies [47, 78, 83]. Most GPs in 1 study from South Africa reported difficulties in referring patients for specialized services [114]; however, this was not an issue for the majority of the GPs from Canada and Sweden [70, 112]. Only 35% of the GPs in 1 UK-based study agreed that there is adequate support for GPs from specialized alcohol services [61]. Better co-operation with specialized services [20, 39, 59, 60, 80], involving other professionals in general practice (e.g. an addiction consultant or a specialized nurse) [39, 71, 105], public health educational campaigns [23, 47, 78, 99, 110] and more media attention [39, 110] were among the most commonly cited facilitators.

##### Theme: Resources

GPs from several countries reported lack of resources for implementing alcohol SBI. Lack of resources included lack of screening tools [20, 23, 47, 74, 78], counselling materials [20, 23, 42, 47, 78, 109] and specific guidelines [103]. Having these resources and displaying information in the waiting room (e.g. posters) were reported as facilitators in several studies [20, 23, 37, 39, 47, 55, 78, 92].

Nine other less frequently mentioned themes were linked to this TDF domain (Table 5).

#### Social influences—TDF domain no. 12

##### Theme: Patients’ feelings when asked about their drinking

Both GPs and nurses in 6 qualitative studies expressed their concern about negative reactions from patients when discussing alcohol issues [49, 84, 86, 89, 92, 105]. However, the majority of both doctors and nurses mentioned that this is the exception rather than the rule in 3 out of 4 studies [70, 73, 95, 102]. Experience with SBI could act as a facilitator.

##### Theme: Doctors’ and nurses’ permissiveness towards alcohol

Some GPs recognized that they have liberal attitudes towards alcohol. In 1 qualitative study from Finland it was pointed out that GPs are members of the community and that it is only natural that they have the same attitudes towards alcohol as their patients [41]. In 2 qualitative studies from the UK and the USA, nurses reported that societal acceptance of heavy drinking can make them hesitate to assess for alcohol in their patients [89, 115].

Six other less frequently mentioned themes were linked to this TDF domain (Table 5).

## Discussion

This review identified a range of barriers and facilitators influencing GPs’ and primary care nurses’ routine delivery of alcohol SBI in adults that linked to the capability, opportunity and motivation components of the COM-B system and to all TDF domains.

The analysis linked all the TDF domains within each component of the COM-B system to at least one of the barriers identified. This suggests that increasing all aspects of capability, opportunity and motivation may be needed for successfully implementing alcohol SBI in primary health care. Furthermore, several barriers linked to more than one TDF domain suggesting that multicomponent strategies may be needed to address some barriers. For example, ‘time’ linked to the TDF domains ‘environmental context and resources’ and ‘beliefs about capabilities’. Restructuring the environment (e.g. involving receptionists in the screening process, arranging for more time per consultation) and modelling (e.g. demonstrating that advising at-risk drinkers within the time of the consultation is manageable) are examples of strategies that could be used to address this barrier. These findings highlight the challenges researchers face in studying alcohol SBI implementation in PHC and help to understand why routine delivery of alcohol SBI in PHC has been proven difficult to implement.

The analysis identified the following TDF domains as having the highest number of data units coded: ‘Environmental Context and Resources’; ‘Beliefs about Capabilities’; and ‘Skills’. Comparatively, few data units were linked to ‘Behaviour Control’, ‘Memory, Attention and Decision Processes’ and ‘Optimism’. Caution should be exerted when deciding the domains on which to intervene based on the frequency a particular barrier is reported in the literature. The behaviour change theories most commonly used in research to explain healthcare professionals’ behaviours are based on constructs related to the reflective, rather than the automatic, aspect of behaviour, which could lead to a bias in the frequency of the reported factors to behaviour change [117]. For example, the majority of studies found in this review are survey-based which provided GPs and nurses with a list of potential barriers, potentially inflating the salience of those barriers whilst neglecting others that could explain the variance of the behaviour. Therefore, it is conceivable that significant barriers to implementation linked to TDF domains with fewer data units coded are yet to be identified, which could give the misleading idea that addressing these domains are less likely to influence implementation. Huijg and colleagues developed a TDF-based questionnaire [118] that could be tailored to study these under-explored barriers and assess their importance.

In a previous review, Johnson and colleagues identified barriers and facilitators to implementing alcohol screening and brief interventions [27]. This review included studies from settings other than PHC, reported only on studies published in English, and gave priority to studies judged most likely to inform UK practice. Our review updates the Johnson et al. review concerning the barriers and facilitators to implementation in PHC. Firstly, we provide evidence on barriers and facilitators from several countries that were not limited to inform a particular practice. One recently published survey study conducted in the largest five European Union countries found that the most frequently cited barriers to implementing alcohol screening among patients with hypertension varied substantially from country to country [119]. This shows that the barriers to, and facilitators of, implementation can vary substantially, between countries that are in geographic proximity and even from place to place within countries. Country features such as individually paid vs nationally funded healthcare, educational level and buying power could influence the salience of a particular factor in the implementation efforts. Therefore, the successful implementation of alcohol SBI will be contingent to tailoring the intervention to local needs [120]. By providing a breakdown by country of study, this review could be of use in the selection of the barriers and facilitators that are more meaningful locally. Secondly, this review was informed by a theoretical framework of behaviour change. Most programmes in practice and research have lacked a theoretical rationale for how they would change practitioner behaviour [28, 121, 122]. Understanding how identified barriers and facilitators fit with the theoretical understandings of behaviour change are key to inform intervention design, and may increase the chances of successful implementation. For example, we have used the results of this review to inform the design of a novel practitioner intervention which has been trialed in Portugal [123]. Therefore, this review may also support researchers in the design of novel theory-based interventions.

### Implications for the implementation of alcohol SBI

Notwithstanding the above-mentioned requirement to tailor the intervention to local needs, mapping the barriers to the components of the COM-B system and domains of the TDF framework allowed for the identification of several content themes that may prove useful in the design of future interventions. Therefore, four key recommendations are suggested based on the results of this review:

1. To develop training programmes for PHC staff

Both GPs and nurses identified lack of knowledge and skills as hindering factors for the routine delivery of alcohol SBI. Examples of issues that need to be considered in training programmes include the following: lack of familiarity with risky drinking guidelines, difficulties in defining low-risk drinking limits, difficulties in differentiating between harmful drinking and alcohol dependence, not knowing how to identify asymptomatic at-risk drinkers, unawareness of standardized screening tools and not knowing how to deliver a brief intervention are. Training could also be designed to address providers’ motivational issues such as lack of confidence in their ability to deliver alcohol SBI, low self-efficacy, believing that patients would resent being asked about alcohol and lack of time;

2. To improve practice organization for preventive counselling

Several GPs reported that PHC practices lack systematic strategies for identifying and advising at-risk drinkers. Strategies for improving practice organization could include involving receptionists in the screening process, having nurses screening for and/or advising at-risk drinkers, and having simple to use screening tools implemented in frequently used questionnaires or registration systems;

3. To provide PHC practices with materials for delivering SBI

GPs commonly reported that a lack of materials for delivering alcohol SBI is an important barrier. Providing PHC practices with guidelines, screening and advice tools and other materials for patients (e.g. posters to display in the waiting room, self-help booklets) are examples of enabling factors to routine alcohol SBI delivery;

4. To involve key stakeholders in the implementation process

Many GPs and nurses reported they were not working in a supportive environment for SBI delivery. Involving PHC management, policy makers, specialized health services, media and available community resources could be key for a successful implementation of alcohol SBI in practice.

### Recommendations for future research

The majority of the studies reported GPs views towards the implementation of alcohol SBI. The views of the nurses are less well studied, although they are regarded as an underutilized resource for implementing alcohol SBI. Future primary research could endeavour to better characterize the barriers and facilitators nurses face when implementing alcohol SBI in PHC.

The majority of the studies retrieved pertain to high-income countries which means that the results of this review may not be representative of barriers and facilitators in lower-income countries.

### Strengths and limitations

The inclusion of both quantitative and qualitative studies from the onset of literature is a strength of this review as it provides a comprehensive understanding of the factors that influence the implementation of alcohol SBI in PHC. This does not mean that all barriers and facilitators will be relevant to all settings; implementation researchers should consider and consult on what makes sense locally. Another strength of this review is that no limitation was applied to the countries in which the study was conducted and a breakdown by country is provided. This allows researchers to directly use data from their own countries and/or to use data from countries they judge to be meaningful locally. A final strength of this review is that it was informed by a theoretical framework to guide the understanding of the barriers and facilitators. We were able to link all extracted data to the components of the COM-B system and TDF domains, providing a well-established structure to support the design of interventions for implementing alcohol SBI in PHC.

A limitation of this review is that it identified barriers and facilitators from the perspective of GPs and nurses only. GPs and nurses often cited the need to involve other PHC staff (e.g. receptionists) in the implementation efforts. Hence, knowing the views of other PHC professionals, management and patients could have been important for a thorough understanding of the factors influencing implementation. This review was limited to studies published in English, French, Portuguese and Spanish: the results do not capture factors from studies which may be published in other languages. We have not taken into account the quality of the studies included in the review whilst synthesizing the findings. However, we report our appraisal of the quality of each study (Table 1) to assist the reader in interpreting the findings. Finally, we limited our search to four databases. Other factors may emerge from searching in other databases and grey literature.

## Conclusion

This study identified a wide range of potential barriers and facilitators to the implementation of alcohol SBI delivery in primary care practices and adds to the scarce body of literature that identifies the barriers and facilitators from a theoretical perspective. Given that alcohol SBI is seldom implemented, this review provides researchers with a tool for designing novel theory-oriented interventions to support the implementation of such activity.

## Supplementary Information

**Additional file 1.** PRISMA 2009 Checklist. This file provides a completed version of the PRISMA Checklist.

**Additional file 2.** Electronic search strategy for the retrieval of studies from multiple databases. This file details the search strategy employed in the review.

**Additional file 3.** List of unobtainable full-text papers. This file details the articles that were selected for full-text analysis but that were not possible to obtain.

**Additional file 4.** Excluded full-text articles and references. This file details the articles that were excluded after full-text analysis.

**Additional file 5.** Title of data: Themes of barriers within each of the components of the COM-B system and domains of the Theoretical Domains Framework. This file details the barriers extracted after full-text analysis.

**Additional file 6.** Facilitators linked to the identified themes of barriers. This file details the facilitators extracted after full-text analysis.

## Data Availability

All data generated or analysed during this study are included in this published article (and its supplementary information files).

## Abbreviations

BCW: Behaviour change wheel
COM-B: Capability-Opportunity-Motivation-Behaviour
GPs: General practitioners
PHC: Primary health care
SBI: Screening and brief interventions
TDF: Theoretical Domains Framework
