# Supplementary material for: Factors influencing the implementation of screening and brief interventions for alcohol use in primary care practices: a systematic review using the COM-B system and Theoretical Domains Framework
Source: Implement Sci. 2021 Jan 7;16:6. doi: 10.1186/s13012-020-01073-0 (PMC7791720; doi:10.1186/s13012-020-01073-0)
Supplement: Supplementary file 2 — Additional file 2. Electronic search strategy for the retrieval of studies from multiple databases. This file details the search strategy employed in the review. [file 13012_2020_1073_MOESM2_ESM.docx]

Additional File 2 – Electronic search strategy for the retrieval of studies from multiple databases

|  | **Databases** | **Search strategy** | |
| --- | --- | --- | --- |
|  | Medline search strategy search = ___ | 1 | advice.tw. |
|  |  | 2 | Attitude of Health Personnel/ |
|  |  | 3 | (behavio?r* adj1 chang*).tw. |
|  |  | 4 | (brief adj advice).tw. |
|  |  | 5 | (brief adj intervention*).tw. |
|  |  | 6 | Cognitive Therapy/ |
|  |  | 7 | (cognitive adj therap*).tw. |
|  |  | 8 | Counseling/ |
|  |  | 9 | counsel*.tw. |
|  |  | 10 | detection.tw. |
|  |  | 11 | exp Directive Counseling/ |
|  |  | 12 | (early adj1 identif*).tw. |
|  |  | 13 | (early adj1 intervention*).tw. |
|  |  | 14 | Health Communication/ |
|  |  | 15 | Health Promotion/ |
|  |  | 16 | identification.tw. |
|  |  | 17 | implementation.tw. |
|  |  | 18 | Interviews as Topic/ |
|  |  | 19 | Mass Screening/ |
|  |  | 20 | Medical History Taking/ |
|  |  | 21 | (minimal adj intervention*).tw. |
|  |  | 22 | (motivat* adj intervention*).tw. |
|  |  | 23 | (motivat* adj interview*).tw. |
|  |  | 24 | Patient Education as Topic/ |
|  |  | 25 | Physician-Patient Relations/ |
|  |  | 26 | Nurse-Patient Relations/ |
|  |  | 27 | Physician's Practice Patterns/ |
|  |  | 28 | Physician's Role/ |
|  |  | 29 | Practice Patterns, Nurses'/ |
|  |  | 30 | Practice Patterns, Physicians'/ |
|  |  | 31 | exp Psychotherapy/ |
|  |  | 32 | Nurse's Role/ |
|  |  | 33 | screening.tw. |
|  |  | 34 | Secondary Prevention/ |
|  |  | 35 | (secondary adj prevention).tw. |
|  |  | 36 | Substance Abuse Detection/ |
|  |  | 37 | "Surveys and Questionnaires"/ |
|  |  | 38 | 1 or 2 or 3 or 4 or 5 or 6 or 7 or 8 or 9 or 10 or 11 or 12 or 13 or 14 or 15 or 16 or 17 or 18 or 19 or 20 or 21 or 22 or 23 or 24 or 25 or 26 or 27 or 28 or 29 or 30 or 31 or 32 or 33 or 34 or 35 or 36 or 37 |
|  |  | 39 | abuse.tw. |
|  |  | 40 | addiction.tw. |
|  |  | 41 | (alcohol* adj1 dependen*).tw. |
|  |  | 42 | exp Alcohol drinking/ |
|  |  | 43 | (alcohol* adj1 drinking).tw. |
|  |  | 44 | (alcohol* adj2 problem*).tw. |
|  |  | 45 | Alcohol-Induced Disorders/ |
|  |  | 46 | Alcohol-Related Disorders/ |
|  |  | 47 | Alcoholic Intoxication/ |
|  |  | 48 | alcoholism.tw. |
|  |  | 49 | Alcoholism/ |
|  |  | 50 | (at-risk adj1 drink*).tw. |
|  |  | 51 | Binge Drinking/ |
|  |  | 52 | (bing* adj drink*).tw. |
|  |  | 53 | dependence.tw. |
|  |  | 54 | drinker*.tw. |
|  |  | 55 | Drinking Behavior/ |
|  |  | 56 | (drink* adj behavio?r*).tw. |
|  |  | 57 | Drinking/ |
|  |  | 58 | (excessiv* adj1 drink*).tw. |
|  |  | 59 | (harmful* adj1 drink*).tw. |
|  |  | 60 | (hazardous adj1 drink*).tw. |
|  |  | 61 | misus*.tw. |
|  |  | 62 | (problem* adj1 drink*).tw. |
|  |  | 63 | (risk* adj1 drink*).tw. |
|  |  | 64 | 39 or 40 or 41 or 42 or 43 or 44 or 45 or 46 or 47 or 48 or 49 or 50 or 51 or 52 or 53 or 54 or 55 or 56 or 57 or 58 or 59 or 60 or 61 or 62 or 63 |
|  |  | 65 | Education, medical/ |
|  |  | 66 | Education, medical, continuing/ |
|  |  | 67 | Education, nursing/ |
|  |  | 68 | Education, nursing, continuing/ |
|  |  | 69 | (family adj doctor*).tw. |
|  |  | 70 | (family adj medicine).tw. |
|  |  | 71 | Family Nurse Practitioners/ |
|  |  | 72 | Family Practice/ |
|  |  | 73 | (family adj practice*).tw. |
|  |  | 74 | General Practice/ |
|  |  | 75 | (general adj practice*).tw. |
|  |  | 76 | General Practitioners/ |
|  |  | 77 | (general adj practitioner*).tw. |
|  |  | 78 | Health Personnel/ed [Education] |
|  |  | 79 | Nurses/ |
|  |  | 80 | Nurse Practitioners/ |
|  |  | 81 | (practice adj nurse*).tw. |
|  |  | 82 | Physicians, Primary Care/ |
|  |  | 83 | Physicians, Family/ |
|  |  | 84 | Physicians/ |
|  |  | 85 | Primary Health Care/ |
|  |  | 86 | (primary adj care).tw. |
|  |  | 87 | (primary adj health*).tw. |
|  |  | 88 | 65 or 66 or 67 or 68 or 69 or 70 or 71 or 72 or 73 or 74 or 75 or 76 or 77 or 78 or 79 or 80 or 81 or 82 or 83 or 84 or 85 or 86 or 87 |
|  |  | 89 | 38 and 64 and 88 |
|  | CINAHL search strategy search = ___ | 1 | TI (advice) OR AB (advice) |
|  |  | 2 | (MH "Attitude of Health Personnel") OR (MH "Physician Attitudes") OR (MH "Nurse Attitudes") |
|  |  | 3 | TI (behavio#r* N1 chang*) OR AB (behavio#r* N1 chang*) |
|  |  | 4 | TI (brief N1 advice) OR AB (brief N1 advice) |
|  |  | 5 | TI (brief N1 intervention*) OR AB (brief N1 intervention*) |
|  |  | 6 | (MH "Cognitive Therapy") |
|  |  | 7 | TI (cognitive N1 therap*) OR AB (cognitive N1 therap*) |
|  |  | 8 | (MH "Counseling") |
|  |  | 9 | TI (counsel*) OR AB (counsel*) |
|  |  | 10 | TI (detection) OR AB (detection) |
|  |  | 11 | TI (early N1 identif*) OR AB (early N1 identif*) |
|  |  | 12 | TI (early N1 intervention*) OR AB (early N1 intervention*) |
|  |  | 13 | (MH "Health Promotion") |
|  |  | 14 | TI (identification) OR AB (identification) |
|  |  | 15 | TI (implementation) OR AB (implementation) |
|  |  | 16 | (MH "Interviews+") |
|  |  | 17 | (MH "Health Screening+") |
|  |  | 18 | (MH "Patient Assessment") OR (MH "Nursing Assessment") |
|  |  | 19 | (MH "Patient History Taking") |
|  |  | 20 | TI (minimal N1 intervention*) OR AB (minimal N1 intervention*) |
|  |  | 21 | TI (motivat* N1 intervention*) OR AB (motivat* N1 intervention*) |
|  |  | 22 | TI (motivat* N1 interview*) OR AB (motivat* N1 interview*) |
|  |  | 23 | (MH "Motivational Interviewing") |
|  |  | 24 | (MH "Patient Education") |
|  |  | 25 | (MH "Professional-Patient Relations") OR (MH "Physician-Patient Relations") |
|  |  | 26 | MH "Nurse-Patient Relations" |
|  |  | 27 | (MH "Physician's Role") |
|  |  | 28 | (MH "Practice Patterns") |
|  |  | 29 | (MH "Psychotherapy+") |
|  |  | 30 | (MH "Nursing Role") |
|  |  | 31 | TI (screening) OR AB (screening) |
|  |  | 32 | (MH "Recurrence/PC") |
|  |  | 33 | TI (secondary N1 prevention) OR AB (secondary N1 prevention) |
|  |  | 34 | (MH "Substance Abuse Detection+") |
|  |  | 35 | (MH "Surveys") OR (MH "Questionnaires+") |
|  |  | 36 | S1 OR S2 OR S3 OR S4 OR S5 OR S6 OR S7 OR S8 OR S9 OR S10 OR S11 OR S12 OR S13 OR S14 OR S15 OR S16 OR S17 OR S18 OR S19 OR S20 OR S21 OR S22 OR S23 OR S24 OR S25 OR S26 OR S27 OR S28 OR S29 OR S30 OR S31 OR S32 OR S33 OR S34 OR S35 |
|  |  | 37 | TI (abuse) OR AB (abuse) |
|  |  | 38 | TI (addiction) OR AB (addiction) |
|  |  | 39 | TI (alcohol* N1 dependen*) OR AB (alcohol* N1 dependen*) |
|  |  | 40 | (MH "Alcohol Abuse") |
|  |  | 41 | (MH "Alcohol Drinking+") |
|  |  | 42 | TI (alcohol* N1 drinking) OR AB (alcohol* N1 drinking) |
|  |  | 43 | TI (alcohol* N2 problem*) OR AB (alcohol* N2 problem*) |
|  |  | 44 | (MH "Alcohol-Induced Disorders, Nervous System") |
|  |  | 45 | (MH "Alcohol-Related Disorders+") |
|  |  | 46 | (MH "Alcoholic Intoxication+") |
|  |  | 47 | (MH "Alcoholics") |
|  |  | 48 | TI (alcoholism) OR AB (alcoholism) |
|  |  | 49 | (MH "Alcoholism") |
|  |  | 50 | TI (at-risk N1 drink*) OR AB (at-risk N1 drink*) |
|  |  | 51 | (MH "Binge Drinking") |
|  |  | 52 | TI (bing* N1 drink*) OR AB (bing* N1 drink*) |
|  |  | 53 | TI (dependence) OR AB (dependence) |
|  |  | 54 | TI (drinker*) OR AB (drinker*) |
|  |  | 55 | MH (Drinking Behavior) |
|  |  | 56 | TI (drink* N1 behavio#r*) OR AB (drink* N1 behavio#r*) |
|  |  | 57 | TI (excessiv* N1 drink*) OR AB (excessiv* N1 drink*) |
|  |  | 58 | TI (harmful* N1 drink*) OR AB (harmful* N1 drink*) |
|  |  | 59 | TI (hazardous N1 drink*) OR AB (hazardous N1 drink*) |
|  |  | 60 | TI (misus*) OR AB (misus*) |
|  |  | 61 | TI (problem* N1 drink*) OR AB (problem* N1 drink*) |
|  |  | 62 | TI (risk* N1 drink*) OR AB (risk* N1 drink*) |
|  |  | 63 | S37 OR S38 OR S39 OR S40 OR S41 OR S42 OR S43 OR S44 OR S45 OR S46 OR S47 OR S48 OR S49 OR S50 OR S51 OR S52 OR S53 OR S54 OR S55 OR S56 OR S57 OR S58 OR S59 OR S60 OR S61 OR S62 |
|  |  | 64 | (MH "Education, Medical+") |
|  |  | 65 | (MH "Education, Medical, Continuing") |
|  |  | 66 | (MH "Education, Nursing+") |
|  |  | 67 | (MH "Education, Nursing, Continuing") |
|  |  | 68 | TI (family N1 doctor*) OR AB (family N1 doctor*) |
|  |  | 69 | TI (family N1 medicine) OR AB (family N1 medicine) |
|  |  | 70 | (MH "Family Nurse Practitioners") |
|  |  | 71 | (MH "Family Practice") |
|  |  | 72 | TI (family N1 practice*) OR AB (family N1 practice*) |
|  |  | 73 | TI (general N1 practice*) OR AB (general N1 practice*) |
|  |  | 74 | TI (general N1 practitioner*) OR AB (general N1 practitioner*) |
|  |  | 75 | (MH "Health Personnel/ED") |
|  |  | 76 | (MH "Nurses") |
|  |  | 77 | (MH "Nurse Practitioners+") |
|  |  | 78 | TI (practice N1 nurse*) OR AB (practice N1 nurse*) |
|  |  | 79 | (MH "Physicians, Family") |
|  |  | 80 | (MH "Physicians") |
|  |  | 81 | (MH "Primary Health Care") |
|  |  | 82 | TI (primary N1 care) OR AB (primary N1 care) |
|  |  | 83 | TI (primary N1 health*) OR AB (primary N1 health*) |
|  |  | 84 | S64 OR S65 OR S66 OR S67 OR S68 OR S69 OR S70 OR S71 OR S72 OR S73 OR S74 OR S75 OR S76 OR S77 OR S78 OR S79 OR S80 OR S81 OR S82 OR S83 |
|  |  | 85 | S36 AND S63 AND S84 |
|  | PsycINFO search strategy search = ___ | 1 | TI (advice) OR AB (advice) |
|  |  | 2 | (DE "Health Personnel Attitudes") |
|  |  | 3 | TI (behavio#r* N1 chang*) OR AB (behavio#r* N1 chang*) |
|  |  | 4 | TI (brief N1 advice) OR AB (brief N1 advice) |
|  |  | 5 | TI (brief N1 intervention*) OR AB (brief N1 intervention*) |
|  |  | 6 | (DE "Cognitive Therapy") |
|  |  | 7 | TI (cognitive N1 therap*) OR AB (cognitive N1 therap*) |
|  |  | 8 | (DE "Counseling") |
|  |  | 9 | TI (counsel*) OR AB (counsel*) |
|  |  | 10 | TI (detection) OR AB (detection) |
|  |  | 11 | TI (early N1 identif*) OR AB (early N1 identif*) |
|  |  | 12 | TI (early N1 intervention*) OR AB (early N1 intervention*) |
|  |  | 13 | (DE "Health Promotion") |
|  |  | 14 | TI (identification) OR AB (identification) |
|  |  | 15 | TI (implementation) OR AB (implementation) |
|  |  | 16 | (DE "Interviews") OR (DE "Interview Schedules") |
|  |  | 17 | DE "Health Screening" OR DE "Physical Examination" |
|  |  | 18 | (DE "Patient History") |
|  |  | 19 | TI (minimal N1 intervention*) OR AB (minimal N1 intervention*) |
|  |  | 20 | TI (motivat* N1 intervention*) OR AB (motivat* N1 intervention*) |
|  |  | 21 | TI (motivat* N1 interview*) OR AB (motivat* N1 interview*) |
|  |  | 22 | (DE "Motivational Interviewing") |
|  |  | 23 | (DE "Client Education") |
|  |  | 24 | (DE "Therapeutic Processes") |
|  |  | 25 | (DE "Professional Role") |
|  |  | 26 | (DE "Health Care Delivery") |
|  |  | 27 | (DE "Psychotherapy" OR DE "Adlerian Psychotherapy" OR DE "Adolescent Psychotherapy" OR DE "Affirmative Therapy" OR DE "Analytical Psychotherapy" OR DE "Autogenic Training" OR DE "Behavior Therapy" OR DE "Brief Psychotherapy" OR DE "Brief Relational Therapy" OR DE "Child Psychotherapy" OR DE "Client Centered Therapy" OR DE "Cognitive Behavior Therapy" OR DE "Conversion Therapy" OR DE "Eclectic Psychotherapy" OR DE "Emotion Focused Therapy" OR DE "Existential Therapy" OR DE "Experiential Psychotherapy" OR DE "Expressive Psychotherapy" OR DE "Eye Movement Desensitization Therapy" OR DE "Feminist Therapy" OR DE "Geriatric Psychotherapy" OR DE "Gestalt Therapy" OR DE "Group Psychotherapy" OR DE "Guided Imagery" OR DE "Humanistic Psychotherapy" OR DE "Hypnotherapy" OR DE "Individual Psychotherapy" OR DE "Insight Therapy" OR DE "Integrative Psychotherapy" OR DE "Interpersonal Psychotherapy" OR DE "Logotherapy" OR DE "Narrative Therapy" OR DE "Network Therapy" OR DE "Persuasion Therapy" OR DE "Primal Therapy" OR DE "Psychoanalysis" OR DE "Psychodrama" OR DE "Psychodynamic Psychotherapy" OR DE "Psychotherapeutic Counseling" OR DE "Rational Emotive Behavior Therapy" OR DE "Reality Therapy" OR DE "Relationship Therapy" OR DE "Solution Focused Therapy" OR DE "Supportive Psychotherapy" OR DE "Transactional Analysis") |
|  |  | 28 | TI (screening) OR AB (screening) |
|  |  | 29 | (DE "Relapse Prevention") |
|  |  | 30 | TI (secondary N1 prevention) OR AB (secondary N1 prevention) |
|  |  | 31 | DE "Drug Usage Screening" |
|  |  | 32 | DE "Questionnaires" OR DE "General Health Questionnaire" OR DE "Surveys" |
|  |  | 33 | S1 OR S2 OR S3 OR S4 OR S5 OR S6 OR S7 OR S8 OR S9 OR S10 OR S11 OR S12 OR S13 OR S14 OR S15 OR S16 OR S17 OR S18 OR S19 OR S20 OR S21 OR S22 OR S23 OR S24 OR S25 OR S26 OR S27 OR S28 OR S29 OR S30 OR S31 OR S32 |
|  |  | 34 | TI (abuse) OR AB (abuse) |
|  |  | 35 | TI (addiction) OR AB (addiction) |
|  |  | 36 | TI (alcohol* N1 dependen*) OR AB (alcohol* N1 dependen*) |
|  |  | 37 | DE "Alcohol Abuse" |
|  |  | 38 | DE "Alcohol Drinking Patterns" OR DE "Social Drinking" |
|  |  | 39 | TI (alcohol* N1 drinking) OR AB (alcohol* N1 drinking) |
|  |  | 40 | TI (alcohol* N2 problem*) OR AB (alcohol* N2 problem*) |
|  |  | 41 | (DE "Alcoholic Psychosis" OR DE "Alcoholic Hallucinosis") OR DE "Fetal Alcohol Syndrome" OR DE "Cirrhosis (Liver)") |
|  |  | 42 | DE "Alcohol Intoxication" OR DE "Chronic Alcoholic Intoxication" OR DE "Acute Alcoholic Intoxication" |
|  |  | 43 | TI (alcoholism) OR AB (alcoholism) |
|  |  | 44 | DE "Alcoholism" |
|  |  | 45 | TI (at-risk N1 drink*) OR AB (at-risk N1 drink*) |
|  |  | 46 | DE "Binge Drinking" |
|  |  | 47 | TI (bing* N1 drink*) OR AB (bing* N1 drink*) |
|  |  | 48 | TI (dependence) OR AB (dependence) |
|  |  | 49 | TI (drinker*) OR AB (drinker*) |
|  |  | 50 | DE "Drinking Behavior" |
|  |  | 51 | TI (drink* N1 behavio#r*) OR AB (drink* N1 behavio#r*) |
|  |  | 52 | TI (excessiv* N1 drink*) OR AB (excessiv* N1 drink*) |
|  |  | 53 | TI (harmful* N1 drink*) OR AB (harmful* N1 drink*) |
|  |  | 54 | TI (hazardous N1 drink*) OR AB (hazardous N1 drink*) |
|  |  | 55 | TI (misus*) OR AB (misus*) |
|  |  | 56 | TI (problem* N1 drink*) OR AB (problem* N1 drink*) |
|  |  | 57 | TI (risk* N1 drink*) OR AB (risk* N1 drink*) |
|  |  | 58 | S34 OR S35 OR S36 OR S37 OR S38 OR S39 OR S40 OR S41 OR S42 OR S43 OR S44 OR S45 OR S46 OR S47 OR S48 OR S49 OR S50 OR S51 OR S52 OR S53 OR S54 OR S55 OR S56 OR S57 |
|  |  | 59 | DE "Medical Education" OR DE "Medical Internship" OR DE "Medical Residency" OR DE "Psychiatric Training" |
|  |  | 60 | DE "Continuing Education" |
|  |  | 61 | DE "Nursing Education" |
|  |  | 62 | TI (family N1 doctor*) OR AB (family N1 doctor*) |
|  |  | 63 | TI (family N1 medicine) OR AB (family N1 medicine) |
|  |  | 64 | TI (family N1 practice*) OR AB (family N1 practice*) |
|  |  | 65 | TI (general N1 practice*) OR AB (general N1 practice*) |
|  |  | 66 | DE "General Practitioners" |
|  |  | 67 | TI (general N1 practitioner*) OR AB (general N1 practitioner*) |
|  |  | 68 | (DE "Nurses") |
|  |  | 69 | TI (practice N1 nurse*) OR AB (practice N1 nurse*) |
|  |  | 70 | (DE "Family Physicians") |
|  |  | 71 | DE "Physicians" |
|  |  | 72 | DE "Primary Health Care" |
|  |  | 73 | TI (primary N1 care) OR AB (primary N1 care) |
|  |  | 74 | TI (primary N1 health*) OR AB (primary N1 health*) |
|  |  | 75 | S59 OR S60 OR S61 OR S62 OR S63 OR S64 OR S65 OR S66 OR S67 OR S68 OR S69 OR S70 OR S71 OR S72 OR S73 OR S74 |
|  |  | 76 | S33 AND S58 AND S75 |
|  | CENTRAL search strategy search = ___ | 1 | "advice" in Trials |
|  |  | 2 | MeSH descriptor: [Attitude of Health Personnel] this term only |
|  |  | 3 | (behavio*r* near/1 chang*) in Trials |
|  |  | 4 | (brief near/1 advice) in Trials |
|  |  | 5 | (brief near/1 intervention*) in Trials |
|  |  | 6 | MeSH descriptor: [Cognitive Therapy] this term only |
|  |  | 7 | (cognitive near/1 therap*) in Trials |
|  |  | 8 | MeSH descriptor: [Counseling] this term only |
|  |  | 9 | (counsel*) in Trials |
|  |  | 10 | (detection) in Trials |
|  |  | 11 | MeSH descriptor: [Directive Counseling] explode all trees |
|  |  | 12 | (early near/1 identif*) in Trials |
|  |  | 13 | (early near/1 intervention*) in Trials |
|  |  | 14 | MeSH descriptor: [Health Communication] this term only |
|  |  | 15 | MeSH descriptor: [Health Promotion] this term only |
|  |  | 16 | (identification) in Trials |
|  |  | 17 | (implementation) in Trials |
|  |  | 18 | MeSH descriptor: [Interviews as Topic] this term only |
|  |  | 19 | MeSH descriptor: [Mass Screening] this term only |
|  |  | 20 | MeSH descriptor: [Medical History Taking] this term only |
|  |  | 21 | (minimal near/1 intervention*) in Trials |
|  |  | 22 | (motivat* near/1 intervention*) in Trials |
|  |  | 23 | (motivat* near/1 interview*) in Trials |
|  |  | 24 | MeSH descriptor: [Patient Education as Topic] this term only |
|  |  | 25 | MeSH descriptor: [Physician-Patient Relations] this term only |
|  |  | 26 | MeSH descriptor: [Nurse-Patient Relations] this term only |
|  |  | 27 | MeSH descriptor: [Practice Patterns, Physicians'] this term only |
|  |  | 28 | MeSH descriptor: [Physician's Role] this term only |
|  |  | 29 | MeSH descriptor: [Practice Patterns, Nurses'] this term only |
|  |  | 30 | MeSH descriptor: [Nurse's Role] this term only |
|  |  | 31 | MeSH descriptor: [Psychotherapy] explode all trees |
|  |  | 32 | (screening) in Trials |
|  |  | 33 | MeSH descriptor: [Secondary Prevention] this term only |
|  |  | 34 | (secondary near/1 prevention) in Trials |
|  |  | 35 | MeSH descriptor: [Substance Abuse Detection] this term only |
|  |  | 36 | MeSH descriptor: [Surveys and Questionnaires] this term only |
|  |  | 37 | #1 or #2 or #3 or #4 or #5 or #6 or #7 or #8 or #9 or #10 or #11 or #12 or #13 or #14 or #15 or #16 or #17 or #18 or #19 or #20 or #21 or #22 or #23 or #24 or #25 or #26 or #27 or #28 or #29 or #30 or #31 or #32 or #33 or #34 or #35 or #36 in Trials |
|  |  | 38 | (abuse) in Trials |
|  |  | 39 | (addiction) in Trials |
|  |  | 40 | (alcohol* near/1 dependen*) in Trials |
|  |  | 41 | MeSH descriptor: [Alcohol Drinking] explode all trees |
|  |  | 42 | (alcohol* near/1 drinking) in Trials |
|  |  | 43 | (alcohol* near/2 problem*) in Trials |
|  |  | 44 | MeSH descriptor: [Alcohol-Induced Disorders] this term only |
|  |  | 45 | MeSH descriptor: [Alcohol-Related Disorders] this term only |
|  |  | 46 | MeSH descriptor: [Alcoholic Intoxication] this term only |
|  |  | 47 | (alcoholism) in Trials |
|  |  | 48 | MeSH descriptor: [Alcoholism] this term only |
|  |  | 49 | (at-risk near/1 drink*) in Trials |
|  |  | 50 | MeSH descriptor: [Binge Drinking] this term only |
|  |  | 51 | (bing* near/1 drink*) in Trials |
|  |  | 52 | (dependence) in Trials |
|  |  | 53 | (drinker*) in Trials |
|  |  | 54 | MeSH descriptor: [Drinking Behavior] this term only |
|  |  | 55 | (drink* near/1 behavio*r*) in Trials |
|  |  | 56 | MeSH descriptor: [Drinking] this term only |
|  |  | 57 | (excessiv* near/1 drink*) in Trials |
|  |  | 58 | (harmful* near/1 drink*) in Trials |
|  |  | 59 | (hazardous near/1 drink*) in Trials |
|  |  | 60 | (misus*) in Trials |
|  |  | 61 | (problem* near/1 drink*) in Trials |
|  |  | 62 | (risk* near/1 drink*) in Trials |
|  |  | 63 | #38 or #39 or #40 or #41 or #42 or #43 or #44 or #45 or #46 or #47 or #48 or #49 or #50 or #51 or #52 or #53 or #54 or #55 or #56 or #57 or #58 or #59 or #60 or #61 or #62 in Trials |
|  |  | 64 | MeSH descriptor: [Education, Medical] this term only |
|  |  | 65 | MeSH descriptor: [Education, Medical, Continuing] this term only |
|  |  | 66 | MeSH descriptor: [Education, Nursing] this term only |
|  |  | 67 | MeSH descriptor: [Education, Nursing, Continuing] this term only |
|  |  | 68 | (family near/1 doctor*) in Trials |
|  |  | 69 | (family near/1 medicine) in Trials |
|  |  | 70 | MeSH descriptor: [Family Nurse Practitioners] this term only |
|  |  | 71 | MeSH descriptor: [Family Practice] this term only |
|  |  | 72 | (family near/1 practice*) in Trials |
|  |  | 73 | MeSH descriptor: [General Practice] this term only |
|  |  | 74 | (general near/1 practice*) in Trials |
|  |  | 75 | MeSH descriptor: [General Practitioners] this term only |
|  |  | 76 | (general near/1 practitioner*) in Trials |
|  |  | 77 | MeSH descriptor: [Health Personnel] this term only and with qualifier(s): [Education - ED] |
|  |  | 78 | MeSH descriptor: [Nurses] this term only |
|  |  | 79 | MeSH descriptor: [Nurse Practitioners] this term only |
|  |  | 80 | (practice near/1 nurse*) in Trials |
|  |  | 81 | MeSH descriptor: [Physicians, Primary Care] this term only |
|  |  | 82 | MeSH descriptor: [Physicians, Family] this term only |
|  |  | 83 | MeSH descriptor: [Physicians] this term only |
|  |  | 84 | MeSH descriptor: [Primary Health Care] this term only |
|  |  | 85 | (primary near/1 care) in Trials |
|  |  | 86 | (primary near/1 health*) in Trials |
|  |  | 87 | #64 or #65 or #66 or #67 or #68 or #69 or #70 or #71 or #72 or #73 or #74 or #75 or #76 or #77 or #78 or #79 or #80 or #81 or #82 or #83 or #84 or #85 or #86 in Trials |
|  |  | 88 | #37 and #63 and #87 in Trials |
