# Supplementary material for: Factors influencing the implementation of screening and brief interventions for alcohol use in primary care practices: a systematic review using the COM-B system and Theoretical Domains Framework
Source: Implement Sci. 2021 Jan 7;16:6. doi: 10.1186/s13012-020-01073-0 (PMC7791720; doi:10.1186/s13012-020-01073-0)
Supplement: Supplementary file 3 — Additional file 3. List of unobtainable full-text papers. This file details the articles that were selected for full-text analysis but that were not possible to obtain. [file 13012_2020_1073_MOESM3_ESM.docx]

Additional file 3. List of unobtainable full-text papers

1. Jones RW, Helrich AR. Treatment of alcoholism by physicians in private practice. A national survey. Quarterly journal of studies on alcohol 1972;33:117-31.

2. Neville RG, Campion PD, Heather N. Barriers to the recognition and management of problem drinking: lessons from a multicentre general practice study. Health bulletin 1987;45:88-94.

3. Weller DP, Litt JC, Pols RG, Ali RL, Southgate DO, Harris RD. Drug and alcohol related health problems in primary care--what do GPs think? The Medical journal of Australia 1992;156:43-8.

4. Roche AM, Richard GP. Early intervention for alcohol problems in general practice: an evaluation of a simple dissemination strategy. Health Promotion Journal of Australia 1994;4:9-12.

5. Leversha AM, Marks RE. Alcohol and pregnancy: doctors' attitudes, knowledge and clinical practice. The New Zealand medical journal 1995;108:428-30.

6. Perdrix A, Decrey H, Pecoud A, Burnand B, Yersin B. [Detection of alcoholism in the medical office: applicability of the CAGE questionnaire by the practicing physician. Group of Medical Practitioners PMU]. Schweizerische medizinische Wochenschrift 1995;125:1772-8.

7. Duszynski KR, Nieto FJ, Valente CM. Reported practices, attitudes, and confidence levels of primary care physicians regarding patients who abuse alcohol and other drugs. Maryland medical journal 1995;44:439-46.

8. Adams PJ, Powell A, McCormick R, Paton-Simpson G. Incentives for general practitioners to provide brief interventions for alcohol problems. The New Zealand medical journal 1997;110:291-4.

9. Davenport TA, Hickie IB, Naismith SL, Hadzi-Pavloviv D, Scott EM. Variability and predictors of mental disorder rates and medical practitioner responses across Australian general practices. The Medical journal of Australia 2001;175 Suppl:S37-41.

10. Peltzer K, Seoka P, Babor T, Obot I. Training primary care nurses to conduct alcohol screening and brief interventions in South Africa. Curationis 2006;29:16-21.

11. Naudet M, Miche JN. (General practice management of alcohol-related problems. Impact of the general practitioner's training and representations). Alcoologie et Addictologie 2006;28:41-50.

12. Michaud P, Fouilland P, Dewost AV, et al. [Early screening and brief intervention among excessive alcohol users: mobilizing general practitioners in an efficient way]. La Revue du praticien 2007;57:1219-26.

13. Hung DY. Improving the delivery of preventive care services. Managed care interface 2007;20:38-44.

14. Souza-Formigoni M, Boemgen-Lacerda R, Vianna V. Implementation of alcohol Screening and brief intervention in primary care units in two Brazilian states: A case study. NAT Nordisk alkohol & narkotikatidskrift 2008;25:533-64.
