# Supplementary material for: Factors influencing the implementation of screening and brief interventions for alcohol use in primary care practices: a systematic review using the COM-B system and Theoretical Domains Framework
Source: Implement Sci. 2021 Jan 7;16:6. doi: 10.1186/s13012-020-01073-0 (PMC7791720; doi:10.1186/s13012-020-01073-0)
Supplement: Supplementary file 4 — Additional file 4. Excluded full-text articles and references. This file details the articles that were excluded after full-text analysis. [file 13012_2020_1073_MOESM4_ESM.docx]

Additional file 4. Excluded full-text articles and references.

**Reasons for exclusion**

1 do not have a title and an abstract

2 is not peer-reviewed and published in an academic journal in the public domain

3 is not published in one of the following languages: English, French, Spanish, or Portuguese

4 do not focus on alcohol

5 do not have a qualitative or quantitative methodology

6 do not focus on the implementation of the intervention in the general primary care adult population

7 do not focus on barriers and/or facilitators reported by general practitioners/family physicians or nurses working in primary care practice

8 Repeated data

**Excluded full-text articles and reasons for exclusion (n=174)**

| First author | Year of publication | Reasons for exclusion |
| --- | --- | --- |
| Aalto | 2000 | 7 |
| Aalto | 2001 | 7 |
| Aalto | 2004 | 7 |
| Aalto | 2005 | 8 |
| Aalto | 2006 | 7 |
| Aalto | 2007 | 7 |
| Abel | 2002 | 7 |
| Agley | 2014 | 7 |
| Allan | 2010 | 7 |
| Amaral | 2010 | 4 |
| Amaral | 2010a | 7 |
| Angove | 2001 | 6 |
| Aspy | 2008 | 7 |
| Assanangkornchai | 2013 | 7 |
| Assanangkornchai | 2014 | 7 |
| Babor | 2004 | 7 |
| Babor | 2005 | 7 |
| Balachova | 2007 | 7 |
| Baldwin | 2006 | 6 |
| Bartek | 1988 | 6 |
| Bendtsen | 1999 | 7 |
| Boekel | 2014 | 6 |
| Brady | 2002 | 5 |
| Brett | 2014 | 7 |
| Bush | 1988 | 7 |
| Campbell-Heider | 2009 | 7 |
| Casswell | 1983 | 8 |
| Chappel | 1977 | 5 |
| Cho | 2003 | 7 |
| Cohen | 1982 | 6 |
| Coogle | 2015 | 7 |
| Copello | 2000 | 6 |
| Costa | 2013 | 4 |
| Crawford-Williams | 2015 | 6 |
| Cruvinel | 2011 | 7 |
| Cruvinel | 2013 | 7 |
| Curry | 2003 | 7 |
| Dunn | 2015 | 4 |
| Dyches | 1999 | 4 |
| Egerer | 2012 | 6 |
| Elwy | 2013 | 7 |
| Ernst | 2007 | 5 |
| Felice | 2012 | 7 |
| Fernald | 2012 | 7 |
| Fleming | 1999 | 5 |
| Fonseca | 2012 | 4 |
| France | 2010 | 7 |
| Furtado | 2008 | 7 |
| Fuste | 2001 | 7 |
| Galanter | 1983 | 7 |
| Garcia | 1991 | 7 |
| Gassman | 2003 | 7 |
| Gassman | 2007 | 7 |
| Gerace | 1995 | 7 |
| Gifford | 2012 | 7 |
| Godlaski | 2012 | 7 |
| Gonçalves | 2011 | 5 |
| Gorman | 1990 | 7 |
| Gottlieb | 1987 | 7 |
| Gray | 1986 | 7 |
| Groves | 2002 | 4 |
| Hanbury | 2015 | 7 |
| Happell | 2002 | 7 |
| Hassoun | 1987 | 7 |
| Heather | 2004 | 7 |
| Herzig | 2006 | 7 |
| Hile | 2003 | 7 |
| Holland | 2009 | 7 |
| Holleman | 2000 | 7 |
| Hore | 1976 | 7 |
| Hung | 2007 | 4 |
| Hunter | 2004 | 7 |
| Hyman | 2010 | 7 |
| Johannessen | 2015 | 7 |
| Johnson | 2005 | 7 |
| Johnson | 2013 | 7 |
| Kaner | 1999a | 7 |
| Kaner | 2001 | 7 |
| Kennedy | 2013 | 7 |
| Kenyon | 2001 | 4 |
| Kessler | 2014 | 4 |
| Ketterer | 2014 | 4 |
| Lamberts | 1999 | 7 |
| Latorre | 2007 | 4 |
| Lev-Ran | 2013 | 7 |
| Linn | 1989 | 7 |
| Linn | 1990 | 7 |
| Linn | 1990a | 7 |
| Livaudais | 2005 | 7 |
| Lock | 2004 | 7 |
| Maciel | 2012 | 7 |
| MacLean | 2013 | 7 |
| Malan | 2015 | 4 |
| Malan | 2015a | 4 |
| Mark | 2003 | 7 |
| Mark | 2003a | 7 |
| Matheson | 2006 | 4 |
| Maynard | 2015 | 4 |
| McCormick | 2010 | 5 |
| McCrady | 1996 | 7 |
| McDaniel | 1989 | 2 |
| McElwaine | 2014 | 7 |
| Mello | 2003 | 7 |
| Mellor | 2013 | 7 |
| Mertens | 2015 | 7 |
| Mignon | 1996 | 7 |
| Miller | 2005 | 5 |
| Moodley-Kunnie | 1988 | 7 |
| Moretti-Pires | 2011 | 7 |
| Mowbray | 1986 | 5 |
| Muench | 2015 | 7 |
| Munro | 2007 | 7 |
| Nalpas | 2003 | 7 |
| Nemeth | 2013 | 7 |
| Neushotz | 2008 | 4 |
| Nilsen | 2011 | 5 |
| Ockene | 1997 | 7 |
| Oliveira | 2012 | 7 |
| Ornstein | 2013 | 7 |
| Panagiotidis | 2010 | 7 |
| Peckover | 2007 | 4 |
| Peltzer | 2008 | 7 |
| Petersen | 2015 | 7 |
| Pillon | 2005 | 7 |
| Poikolainen | 1988 | 7 |
| Potamianos | 1985 | 7 |
| Pursch | 1978 | 5 |
| Rahm | 2015 | 7 |
| Raistrick | 2008 | 7 |
| Richmond | 1994 | 5 |
| Rieckmann | 2010 | 4 |
| Rivers | 1998 | 4 |
| Roberts | 2008 | 4 |
| Robertson | 2015 | 4 |
| Roche | 2001 | 6 |
| Roche | 1991 | 7 |
| Rohman | 1987 | 7 |
| Ronzani | 2005 | 7 |
| Ronzani | 2009 | 7 |
| Rose | 2016 | 7 |
| Rosenstock | 2010 | 4 |
| Rosso | 1992 | 7 |
| Rosta | 2003 | 7 |
| Rowland | 1989 | 7 |
| Rush | 2013 | 4 |
| Saitz | 2002 | 7 |
| Seppannen | 2012 | 7 |
| Sibthorpe | 2002 | 7 |
| Skinner | 2007 | 7 |
| Smith | 2003 | 4 |
| Soares | 2013 | 7 |
| Spandorfer | 1999 | 7 |
| Stockwell | 1990 | 7 |
| Stoner | 2014 | 7 |
| Strang | 2007 | 4 |
| Strayer | 2012 | 7 |
| Tam | 2013 | 7 |
| Taylor | 2007 | 7 |
| Thomas | 2014 | 7 |
| Thompson | 2001 | 4 |
| Tober | 1990 | 5 |
| Tønnesen | 2010 | 7 |
| Townes | 1994 | 7 |
| Urada | 2014 | 4 |
| Vadlamudi | 2008 | 7 |
| van Boekel | 2014 | 4 |
| van Boekel | 2015 | 4 |
| Vargas | 2008 | 7 |
| Vargas | 2010 | 7 |
| Wallston | 1976 | 7 |
| Waring | 1975 | 7 |
| Weinehall | 2014 | 7 |
| Whiteford | 2015 | 7 |
| Williams | 1999 | 7 |

**References of excluded full-text articles**

**Aalto 2000**

Aalto M, Saksanen R, Laine P, Forsström R, Raikaa M, Kiviluoto M et al. Brief intervention for female heavy drinkers in routine general practice: a 3-year randomized, controlled study. Alcoholism: Clinical & Experimental Research 2000; 24(11):1680-1686.

**Aalto 2001**

Aalto M, Seppa K, Mattila P, Mustonen H, Ruuth K, Hyvarinen H et al. Brief intervention for male heavy drinkers in routine general practice: A three-year randomized controlled study. Alcohol and alcoholism 2001; 36(3):224-230.

**Aalto 2004**

Aalto M, Seppa K. Usefulness, length and content of alcohol-related discussions in primary health care: the exit poll survey. Alcohol Alcohol 2004; 39(6):532-535.

**Aalto 2005**

Aalto M, Pekuri P, Seppä K. Implementation of brief alcohol intervention in primary health care: do nurses' and general practitioners' attitudes, skills and knowledge change? Drug Alcohol Rev 2005; 24(6):555-558.

**Aalto 2006**

Aalto M, Hyvönen S, Seppä K. Do primary care physicians' own AUDIT scores predict their use of brief alcohol intervention? A cross-sectional survey. Drug & Alcohol Dependence 2006; 83(2):169-173.

**Aalto 2007**

Aalto M, Seppä K. Primary health care physicians' definitions on when to advise a patient about weekly and binge drinking. Addict Behav 2007; 32(7):1321-1330.

**Abel 2002**

Abel EL, Kruger M. Physician attitudes concerning legal coercion of pregnant alcohol and drug abusers. Am J Obstet Gynecol 2002; 186(4):768-772.

**Agley 2014**

Agley J, McIntire R, DeSalle M, Tidd D, Wolf J, Gassman R. Connecting patients to services: Screening, brief intervention and referral to treatment in primary health care. Drugs: Education, Prevention & Policy 2014; 21(5):370-379.

**Allan 2010**

Allan J. Engaging primary health care workers in drug and alcohol and mental health interventions: challenges for service delivery in rural and remote Australia. Aust J Prim Health 2010; 16(4):311-318.

**Amaral 2010**

Amaral-Sabadini MB, Saitz R, Souza-Formigoni ML. Do attitudes about unhealthy alcohol and other drug (AOD) use impact primary care professionals' readiness to implement AOD-related preventive care? Drug & Alcohol Review 2010; 29(6):655-661.

**Amaral 2010a**

Amaral MB, Ronzani TM, Souza-Formigoni ML. Process evaluation of the implementation of a screening and brief intervention program for alcohol risk in primary health care: An experience in Brazil. Drug & Alcohol Review 2010; 29(2):162-168.

**Angove 2001**

Angove R, McBride AJ. Swimming upstream: how and why an alcohol misuse screening and intervention service using the AUDIT can have limited impact in primary care. Journal of Substance Use 2001; 6(2):70-79.

**Aspy 2008**

Aspy CB, Mold JW, Thompson DM, Blondell RD, Landers PS, Reilly KE et al. Integrating screening and interventions for unhealthy behaviors into primary care practices. Am J Prev Med 2008; 35(5):S373-S380.

**Assanangkornchai 2013**

Assanangkornchai S, Balthip Q, Edwards JG. Screening and brief intervention for substance misuse in Thailand. Public health 2013; 127(12):1140-1142.

**Assanangkornchai 2014**

Assanangkornchai S, Balthip Q, Edwards JG, assistance of the ASSIST-SBI Development Co-investigators. Implementing the Alcohol, Smoking, Substance Involvement Screening Test and linked brief intervention service in primary care in Thailand. J Public Health (Oxf) 2014; 36(3):443-449.

**Babor 2004**

Babor TF, Higgins-Biddle JC, Higgins PS, Gassman RA, Gould BE. Training medical providers to conduct alcohol screening and brief interventions. Subst Abus 2004; 25(1):17-26.

**Babor 2005**

Babor TF, Higgins-Biddle J, Dauser D, Higgins P, Burleson JA. Alcohol Screening and Brief Intervention in Primary Care Settings: Implementation Models and Predictors. J stud alcohol 2005; 66(3):361-368.

**Balachova 2007**

Balachova TN, Bonner BL, Isurina GL, Tsvetkova LA. Use of focus groups in developing FAS/FASD prevention in Russia. Subst Use Misuse 2007; 42(5):881-894.

**Baldwin 2006**

Baldwin JA, Johnson RM, Wayment HA, Callahan EJ. Partnering with community-based professionals to improve substance abuse screening and intervention for youth and young adults. Subst Abus 2006; 26(3/4):43-47.

**Bartek 1988**

Bartek JK, Lindeman M, Newton M, Fitzgerald AP, Hawks JH. Nurse-identified problems in the management of alcoholic patients. J stud alcohol 1988; 49(1):62-70.

**Bendtsen 1999**

Bendtsen P, Akerlind I. Changes in attitudes and practices in primary health care with regard to early intervention for problem drinkers. Alcohol and alcoholism 1999; 34(5):795-800.

**Boekel 2014**

Boekel LC, Brouwers EP, Weeghel J, Garretsen HF. Healthcare professionals' regard towards working with patients with substance use disorders: comparison of primary care, general psychiatry and specialist addiction services. Drug Alcohol Depend 2014; 134:92-98.

**Brady 2002**

Brady M, Sibthorpe B, Bailie R, Ball S, Sumnerdodd P. The feasibility and accaptability of introducing brief intervention for alcohol misuse in an urban Aboriginal medical service. Drug Alcohol Rev 2002; 21(4):375-380.

**Brett 2014**

Brett J, Lawrence L, Ivers R, Conigrave K. Outpatient alcohol withdrawal management for Aboriginal and Torres Strait Islander peoples. Aust Fam Physician 2014; 43(8):563-566.

**Bush 1988**

Bush RA, Williams RJ. Generalist health and welfare workers' response to alcohol related problems: role legitimacy and the need for role-support, an example from an Australian rural community. Drug Alcohol Depend 1988; 22(1-2):105-111.

**Campbell-Heider 2009**

Campbell-Heider N, Finnell DS, Feigenbaum JC, Feeley TH, Rejman KS, Austin-Ketch T et al. Survey on addictions: toward curricular change for family nurse practitioners. Int j nurs educ scholarsh 2009; 6(1):1p-17.

**Casswell 1983**

Casswell S, McPherson M. Attitudes of New Zealand general practitioners to alcohol-related problems. J stud alcohol 1983; 44(2):342-351.

**Chappel 1977**

Chappel JN, Schnoll SH. Physician attitudes. Effect on the treatment of chemically dependent patients. JAMA 1977; 237(21):2318-2319.

**Cho 2003**

Cho HJ, Sunwoo S, Song YM. Attitudes and reported practices of Korean primary care physicians for health promotion. J Korean Med Sci 2003; 18(6):783-790.

**Cohen 1982**

Cohen GH, Griffin PT, Wiltz GM. Stereotyping as a negative factor in substance abuse treatment. International Journal of the Addictions 1982; 17(2):371-376.

**Coogle 2015**

Coogle C, Owens M. Screening and Brief Intervention for Alcohol Misuse in Older Adults: Training Outcomes Among Physicians and Other Healthcare Practitioners in Community-Based Settings. Community Ment Health J 2015; 51(5):546-553.

**Copello 2000**

Copello A, Templeton L, Krishnan M, Orford J, Velleman R. A treatment package to improve primary care services for relatives of people with alcohol and drug problems. Addiction Research 2000; 8(5):471-484.

**Costa 2013**

Costa PHAd, Mota DCB, Cruvinel E, Paiva FSd, Ronzani TM. [A methodology to implement preventive actions against harmful drug use in the context of primary health care in Latin America]. Rev Panam Salud Publica 2013; 33(5):325-331.

**Crawford-Williams 2015**

Crawford-Williams F, Steen M, Esterman A, Fielder A, Mikocka-Walus A. "If you can have one glass of wine now and then, why are you denying that to a woman with no evidence": Knowledge and practices of health professionals concerning alcohol consumption during pregnancy. Women Birth 2015; 28(4):329-335.

**Cruvinel 2011**

Cruvinel E, Ronzani TM. Clima organizacional e atividades de prevenção ao uso de risco de álcool. Estudos de Psicologia 2011; 28(2):209-217.

**Cruvinel 2013**

Cruvinel E, Richter KP, Bastos RR, Ronzani TM. Screening and brief intervention for alcohol and other drug use in primary care: associations between organizational climate and practice. Addict Sci Clin Pract 2013; 8:4.

**Curry 2003**

Curry SJ, Ludman EJ, Grothaus LC, Donovan D, Kim E. A randomized trial of a brief primary-care-based intervention for reducing at-risk drinking practices. Health Psychol 2003; 22(2):156-165.

**Dunn 2015**

Dunn C, Darnell D, Carmel A, Atkins DC, Bumgardner K, Roy-Byrne P. Comparing the motivational interviewing integrity in two prevalent models of brief intervention service delivery for primary care settings. J Subst Abuse Treat 2015; 51:47-52.

**Dyches 1999**

Dyches H, Alemagno S, Llorens SA, Butts JM. Automated telephone-administered substance abuse screening for adults in primary care. Health Care Manag Sci 1999; 2(4):199-204.

**Egerer 2012**

Egerer M. Alcoholism, brief intervention and the institutional context: a focus-group study with French and Finnish general practitioners. Critical Public Health 2012; 22(3):307-318.

**Elwy 2013**

Elwy AR, Horton NJ, Saitz R. Physicians' attitudes toward unhealthy alcohol use and self-efficacy for screening and counseling as predictors of their counseling and primary care patients' drinking outcomes. Substance Abuse Treatment, Prevention & Policy 2013; 8:17.

**Ernst 2007**

Ernst D, Miller WR, Rollnick S. Treating substance abuse in primary care: a demonstration project. Int J Integr Care 2007; 7:e36.

**Felice 2012**

Felice AM, Kouimtsidis C. Improving services offered by GPs to patients with drug addiction. Mental Health Practice 2012; 16(1):19-22.

**Fernald 2012**

Fernald DH, Dickinson LM, Froshaug DB, Balasubramanian BA, Holtrop JS, Krist AH et al. Improving Multiple Health Risk Behaviors in Primary Care: Lessons from the Prescription for Health COmmon Measures, Better Outcomes (COMBO) Study. Journal of the American Board of Family Medicine 2012; 25(5):701-711.

**Fleming 1999**

Fleming M, Manwell LB. Brief intervention in primary care settings: a primary treatment method for at-risk, problem, and dependent drinkers. Alcohol Research & Health 1999; 23(2):128-137.

**Fonseca 2012**

Fonseca F, Gilchrist G, Torrens M. Integrating addiction and mental health networks to improve access to treatment for people with alcohol and drug-related problems: a qualitative study. Advances in Dual Diagnosis 2012; 5(1):5-14.

**France 2010**

France K, Henley N, Payne J, D'Antoine H, Bartu A, O'Leary C et al. Health professionals addressing alcohol use with pregnant women in Western Australia: barriers and strategies for communication. Subst Use Misuse 2010; 45(10):1474-1490.

**Furtado 2008**

Furtado EF, Corradi-Webster CM, Laprega MR. Implementing brief interventions for alcohol problems in the public health system in the region of Ribeirao Preto, Brazil: Evaluation of the PAI-PAD training model. NAT Nordisk alkohol & narkotikatidskrift 2008; 25(6):539-551.

**Fuste 2001**

Fuste J, Rue M. [Variability in preventive activities among primary care teams in Catalonia. Application of a multilevel analysis]. Gac Sanit 2001; 15(2):118-127.

**Galanter 1983**

Galanter M, Blume S, Bissell L. Physicians in alcoholism: A study of current status and future needs. Alcoholism: Clinical and Experimental Research 1983; 7(4):389-392.

**Garcia 1991**

Garcia J, Osuna E, Perez-Carceles MD, Luna A. Attitudes of medical practitioners towards alcohol and alcohol consumption. Med Law 1991; 10(5):469-475.

**Gassman 2003**

Gassman RA. Medical specialization, profession, and mediating beliefs that predict stated likelihood of alcohol screening and brief intervention: targeting educational interventions. Subst Abus 2003; 24(3):141-156.

**Gassman 2007**

Gassman RA. Practitioner-level predictors of alcohol problems detection and management activities. Journal of Substance Use 2007; 12(3):191-202.

**Gerace 1995**

Gerace LM, Hughes TL, Spunt J. Improving nurses' responses toward substance-misusing patients: a clinical evaluation project. Arch Psychiatr Nurs 1995; 9(5):286-294.

**Gifford 2012**

Gifford H, Paton S, Cvitanovic L, McMenamin J, Newton C. Is routine alcohol screening and brief intervention feasible in a New Zealand primary care environment? New Zealand Medical Journal 2012; 125(1354):17-25.

**Godlaski 2012**

Godlaski TM, Clark JJ. Implementation issues in an innovative rural substance misuser treatment program. Subst Use Misuse 2012; 47(13-14):1439-1450.

**Gonçalves 2011**

Gonçalves A, Ferreira P, Abreu A, Pillon S, Jezus S. Estratégias de rastreamento e intervenções breves como possibilidades para a prática preventiva do enfermeiro. Revista Eletrônica de Enfermagem 2011; 13(2):355-360.

**Gorman 1990**

Gorman DM, Werner JM, Jacobs LM, Duffy SW. Evaluation of an alcohol education package for non-specialist health care and social workers. Br J Addict 1990; 85(2):223-233.

**Gottlieb 1987**

Gottlieb NH, Mullen PD, McAlister AL. Patients' substance abuse and the primary care physician: Patterns of practice. Addict Behav 1987; 12(1):23-32.

**Gray 1986**

Gray RH, Smith MC, Garner DD, Cage BN, Freeman RA. Designing new substance abuse treatment services for a competitive environment. J Ment Health Adm 1986; 13(2):15-22.

**Groves 2002**

Groves P, Heuston J, Albery I, Gerada C, Gossop M, Strang J. Evaluation of a service to strengthen primary care responses to substance-misusing patients: Welcomed, but little impact. Drugs: Education, Prevention & Policy 2002; 9(1):21-33.

**Hanbury 2015**

Hanbury A, Farley K, Thompson C, Wilson PM. Assessment of fidelity in an educational workshop designed to increase the uptake of a primary care alcohol screening recommendation. J Eval Clin Pract 2015; 21(5):873-878.

**Happell 2002**

Happell B, Carta B, Pinikahana J. Nurses' knowledge, attitudes and beliefs regarding substance use: A questionnaire survey. Nurs Health Sci 2002; 4(4):193-200.

**Hassoun 1987**

Hassoun J, Labarthe F, Maisondieu J. Medecins et alcooliques: a propos d'une enquete. Annales Médico-Psychologiques 1987; 145(9):794-798.

**Heather 2004**

Heather N, Dallolio E, Hutchings D, Kaner E, White M. Implementing routine screening and brief alcohol intervention in primary health care: a Delphi survey of expert opinion. Journal of Substance Use 2004; 9(2):68-85.

**Herzig 2006**

Herzig K, Huynh D, Gilbert P, Danley DW, Jackson R, Gerbert B. Comparing prenatal providers approaches to four different risks: alcohol, tobacco, drugs, and domestic violence. Women Health 2006; 43(3):83-101.

**Hile 2003**

Hile MG. Mental health and substance abuse screening in primary care. Journal of Technology in Human Services 2003; 21(3):21-34.

**Holland 2009**

Holland CL, Pringle JL, Barbetti V. Identification of physician barriers to the application of screening and brief intervention for problem alcohol and drug use. Alcoholism Treatment Quarterly 2009; 27(2):174-183.

**Holleman 2000**

Holleman MC, Thornby JI, Merrill JM. Substance abusers: Role of personal and professional role traits in caregivers' causal attributions. Psychol Rep 2000; 86(2):407-413.

**Hore 1976**

Hore BD, Wilkins RH. A general-practice study of the commonest presenting symptoms of alcoholism. J R Coll Gen Pract 1976; 26(163):140-142.

**Hung 2007**

Hung DY, Rundall TG, Tallia AF, Cohen DJ, Halpin HA, Crabtree BF. Rethinking prevention in primary care: applying the Chronic Care Model to address health risk behaviors. Milbank Quarterly 2007; 85(1):69-91.

**Hunter 2004**

Hunter E, Brown J, McCulloch B. Encouraging practitioners to use resources: Evaluation of the national implementation of a resource to improve the clinical management of alcohol-related problems in Indigenous primary care settings. Drug Alcohol Rev 2004; 23(1):89-100.

**Hyman 2010**

Hyman Z, Crosby F. Study of implementation of a guideline for brief alcohol intervention in primary care. J Nurs Care Qual 2010; 25(1):46-55.

**Johannessen 2015**

Johannessen A, Engedal K, Helvik AS. Use and misuse of alcohol and psychotropic drugs among older people: Is that an issue when services are planned for and implemented? Scand J Caring Sci 2015; 29(2):325-332.

**Johnson 2005**

Johnson TP, Booth AL, Johnson P. Physician Beliefs About Substance Misuse and Its Treatment: Findings from a U.S. Survey of Primary Care Practitioners. Subst Use Misuse 2005; 40(8):1071-1084.

**Johnson 2013**

Johnson JA, Seale JP, Shellenberger S, Hamrick M, Lott R. Impact of system-level changes and training on alcohol screening and brief intervention in a family medicine residency clinic: a pilot study. Substance Abuse Treatment, Prevention & Policy 2013; 8:9.

**Kaner 1999**

Kaner EF, Lock CA, McAvoy BR, Heather N, Gilvarry E. A RCT of three training and support strategies to encourage implementation of screening and brief alcohol intervention by general practitioners. British Journal of General Practice 1999; 49(446):699-703.

**Kaner 2001**

Kaner EF, Heather N, Brodie J, Lock CA, McAvoy BR. Patient and practitioner characteristics predict brief alcohol intervention in primary care. British Journal of General Practice 2001; 51(471):822-827.

**Kennedy 2013**

Kennedy AJ, Mellor D, McCabe MP, Ricciardelli LA, Brumby SA, Head A et al. Training and experience of nurses in responding to alcohol misuse in rural communities. Public health nursing 2013; 30(4):332-342.

**Kenyon 2001**

Kenyon R, West D, Raistrick D, Hatton P. General practitioner satisfaction with 'shared care' working. Journal of Substance Use 2001; 6(1):36-39.

**Kessler 2014**

Kessler R, Miller BF, Kelly M, Graham D, Kennedy A, Littenberg B et al. Mental health, substance abuse, and health behavior services in patient-centered medical homes. Journal of the American Board of Family Medicine 2014; 27(5):637-644.

**Ketterer 2014**

Ketterer F, Symons L, Lambrechts MC, Mairiaux P, Godderis L, Peremans L et al. What factors determine Belgian general practitioners' approaches to detecting and managing substance abuse? A qualitative study based on the I-Change Model. BMC Fam Pract 2014; 15:119.

**Lamberts 1999**

Lamberts H, Okkes I. Patients with chronic alcohol abuse in Dutch family practices. Alcohol and alcoholism 1999; 34(3):337-345.

**Latorre 2007**

Latorre J, Lopez-Torres J, Sanchez-Nunez T, Serrano JP, Montanes J, Escobar F. Primary care doctors' perception of treatment demand and need for training in drug addiction issues. Primary Care & Community Psychiatry 2007; 12(1):33-41.

**Lev-Ran 2013**

Lev-Ran S, Adler L, Nitzan U, Fennig S. Attitudes towards nicotine, alcohol and drug dependence among physicians in Israel. J Subst Abuse Treat 2013; 44(1):84-89.

**Linn 1989**

Linn LS, Yager J. Factors associated with physician recognition and treatment of alcoholism. West J Med 1989; 150(4):468-472.

**Linn 1990**

Linn LS, Yager J, Leake B. Physicians' attitudes toward substance abuse and drug testing. International Journal of the Addictions 1990; 25(4):427-444.

**Linn 1990a**

Linn LS, Yager J, Leake B. Professional vs. personal factors related to physicians' attitudes toward drug testing. J Drug Educ 1990; 20(2):95-109.

**Livaudais 2005**

Livaudais JC, Kaplan CP, Haas JS, P+®rez-Stable EJ, Stewart S, des JG. Lifestyle behavior counseling for women patients among a sample of California physicians. Journal of Women's Health (15409996) 2005; 14(6):485-495.

**Lock 2004**

Lock CA, Kaner EFS. Implementation of brief alcohol interventions by nurses in primary care: Do non-clinical factors influence practice? Fam Pract 2004; 21(3):270-275.

**Maciel 2012**

Maciel S, Oliveira R, Melo J. Alcoolismo em indígenas potiguara: representações sociais dos profissionais de saúde. Psicologia: Ciência e Profissão 2012; 32(1):98-111.

**MacLean 2013**

MacLean S, Berends L, Mugavin J. Factors contributing to the sustainability of alcohol and other drug interventions in Australian community health settings. Aust J Prim Health 2013; 19(1):53-58.

**Malan 2015**

Malan Z, Mash B, Everett-Murphy K. A situational analysis of training for behaviour change counselling for primary care providers, South Africa. Afr j prim health care fam med 2015; 7(1).

**Malan 2015a**

Malan Z, Mash R, Everett-Murphy K. Qualitative evaluation of primary care providers experiences of a training programme to offer brief behaviour change counselling on risk factors for non-communicable diseases in South Africa. BMC Fam Pract 2015; 16(1):1-10.

**Mark 2003**

Mark TL, Kranzler HR, Song X, Bransberger P, Poole VH, Crosse S. Physicians' opinions about medications to treat alcoholism. Addiction 2003; 98(5):617-626.

**Mark 2003a**

Mark TL, Kranzler HR, Poole VH, Hagen CA, McLeod C, Crosse S. Barriers to the Use of Medications to Treat Alcoholism. The American Journal on Addictions 2003; 12(4):281-294.

**Matheson 2006**

Matheson C, van Teijlingen E, Bond CM, Cameron IM, Cronkshaw G. Specialist substance misuse nurses' assessment and decision making in the management of drug misusers. Drugs: Education, Prevention & Policy 2006; 13(6):551-562.

**Maynard 2015**

Maynard S, Campbell E, Boodhoo K, Gauthier G, Xenocostas S, Charney DA et al. From Policy to Practice: Implementation of Treatment for Substance Misuse in Quebec Primary Healthcare Clinics. Healthc Policy 2015; 11(2):86-101.

**McCormick 2010**

McCormick R, Docherty B, Segura L, Colom J, Gual A, Cassidy P et al. The research translation problem: Alcohol screening and brief intervention in primary care - Real world evidence supports theory. Drugs: Education, Prevention & Policy 2010; 17(6):732-748.

**McCrady 1996**

McCrady BS, Richter SS, Morgan TJ, Slade J, Pfeifer C. Involving health care workers in screening for alcohol problems. J Addict Dis 1996; 15(3):45-58.

**McDaniel 1989**

McDaniel SH, Campbell TL, Seaburn DB. Managing personal and professional boundaries: How to make the physician's own issues a resource in patient care. Family Systems Medicine 1989; 7(4):385-396.

**McElwaine 2014**

McElwaine KM, Freund M, Campbell EM, Slattery C, Wye PM, Lecathelinais C et al. Clinician assessment, advice and referral for multiple health risk behaviors: Prevalence and predictors of delivery by primary health care nurses and allied health professionals. Patient Education & Counseling 2014; 94(2):193-201.

**Mello 2003**

Mello MJ, Nirenberg TD, Lindquist D, Cullen HA, Woolard R. Physicians' attitudes regarding reporting alcohol-impaired drivers. Subst Abus 2003; 24(4):233-242.

**Mellor 2013**

Mellor D, McCabe M, Ricciardelli LA, Brumby S, Head A, Mercer-Grant C et al. Evaluation of an alcohol intervention training program for nurses in rural Australia. Journal of Research in Nursing 2013; 18(6):561-575.

**Mertens 2015**

Mertens JR, Chi FW, Weisner CM, Satre DD, Ross TB, Allen S et al. Physician versus non-physician delivery of alcohol screening, brief intervention and referral to treatment in adult primary care: the ADVISe cluster randomized controlled implementation trial. Addict Sci Clin Pract 2015; 10:26.

**Mignon 1996**

Mignon SI. Physicians' perceptions of alcoholics: The disease concept reconsidered. Alcoholism Treatment Quarterly 1996; 14(4):33-45.

**Miller 2005**

Miller WR, Moyers TB, Arciniega L, Ernst D, Forcehimes A. Training, supervision and quality monitoring of the COMBINE Study behavioral interventions. J Stud Alcohol Suppl 2005;(15):188-189.

**Moodley-Kunnie 1988**

Moodley-Kunnie T. Attitudes and perceptions of health professionals toward substance use disorders and substance-dependent individuals. International Journal of the Addictions 1988; 23(5):469-475.

**Moretti-Pires 2011**

Moretti-Pires RO, Corradi-Webster CM. Implementation of brief intervention for problematic alcohol use in primary health in the amazon context. Revista Latino-Americana de Enfermagem (RLAE) 2011; 19:813-820.

**Mowbray 1986**

Mowbray A, Kessel N. Alcoholism and the general practitioner. The British Journal of Psychiatry 1986; 148:697-700.

**Muench 2015**

Muench J, Jarvis K, Vandersloot D, Hayes M, Nash W, Hardman J et al. Perceptions of Clinical Team Members Toward Implementation of SBIRT Processes. Alcoholism Treatment Quarterly 2015; 33(2):143-160.

**Munro 2007**

Munro A, Watson HE, McFadyen A. Assessing the impact of training on mental health nurses' therapeutic attitudes and knowledge about co-morbidity: a randomised controlled trial. Int J Nurs Stud 2007; 44(8):1430-1438.

**Nalpas 2003**

Nalpas B, Lemaitre R, Dalbies PA, Monod P, Martin S, Balmes JL. [Attitudes and opinions of private practitioners towards alcoholism. A survey in the Languedoc-Roussillon region]. Presse Med 2003; 32(9):391-399.

**Nemeth 2013**

Nemeth LS, Miller PM, Nietert PJ, Ornstein SM, Wessell AM, Jenkins RG. Organizational attributes and screening and brief intervention in primary care. Addict Behav 2013; 38(11):2639-2642.

**Neushotz 2008**

Neushotz LA, Fitzpatrick JJ. Improving substance abuse screening and intervention in a primary care clinic. Arch Psychiatr Nurs 2008; 22(2):78-86.

**Nilsen 2011**

Nilsen P, Wahlin S, Heather N. Implementing brief interventions in health care: lessons learned from the Swedish Risk Drinking Project. Int J Environ Res Public Health 2011; 8(9):3609-3627.

**Ockene 1997**

Ockene JK, Wheeler EV, Adams A, Hurley TG, Hebert J. Provider training for patient-centered alcohol counseling in a primary care setting. Arch Intern Med 1997; 157(20):2334-2341.

**Oliveira 2012**

Oliveira M, Ronzani T. Estigmatização e prática de profissionais da APS referentes ao consumo de álcool. Psicologia: Ciência e Profissão 2012; 32(3):648-661.

**Ornstein 2013**

Ornstein SM, Miller PM, Wessell AM, Jenkins RG, Nemeth LS, Nietert PJ. Integration and sustainability of alcohol screening, brief intervention, and pharmacotherapy in primary care settings. Journal of Studies on Alcohol & Drugs 2013; 74(4):598-604.

**Panagiotidis 2010**

Panagiotidis P, Papadopoulou M, Diakogiannis I. Identification and Brief Interventions for Alcohol Problems: Evaluation of a Training Program for Primary Health Care Professionals. Alcoholism Treatment Quarterly 2010; 28(4):464-479.

**Peckover 2007**

Peckover S, Chidlaw RG. Too frightened to care? Accounts by district nurses working with clients who misuse substances. Health Soc Care Community 2007; 15(3):238-245.

**Peltzer 2008**

Peltzer K, Matseke G, Azwihangwisi M. Evaluation of alcohol screening and brief intervention in routine practice of primary care nurses in Vhembe district, South Africa. Croat Med J 2008; 49(3):392-401.

**Petersen 2015**

Petersen Williams P, Petersen Z, Sorsdahl K, Mathews C, Everett-Murphy K, Parry CD. Screening and Brief Interventions for Alcohol and Other Drug Use Among Pregnant Women Attending Midwife Obstetric Units in Cape Town, South Africa: A Qualitative Study of the Views of Health Care Professionals. J Midwifery Womens Health 2015; 60(4):401-409.

**Pillon 2005**

Pillon SC, Laranjeira RR. Formal education and nurses' attitudes towards alcohol and alcoholism in a Brazilian sample. Sao Paulo Med J 2005; 123(4):175-180.

**Poikolainen 1988**

Poikolainen K. Alcohol-related knowledge, beliefs and attitudes among health and clerical personnel. Social science & medicine 1988; 27(12):1429-1432.

**Potamianos 1985**

Potamianos G, Winter D, Duffy SW, Gorman DM, Peters TJ. The perception of problem drinkers by general hospital staff, general practitioners and alcoholic patients. Alcohol 1985; 2(4):563-566.

**Pursch 1978**

Pursch JA. Physicians' attitudinal changes in alcoholism. Alcohol Clin Exp Res 1978; 2(4):358-361.

**Rahm 2015**

Rahm AK, Boggs JM, Martin C, Price DW, Beck A, Backer TE et al. Facilitators and Barriers to Implementing Screening, Brief Intervention, and Referral to Treatment (SBIRT) in Primary Care in Integrated Health Care Settings. Subst Abus 2015; 36(3):281-288.

**Raistrick 2008**

Raistrick D, Russell D, Tober G, Tindale A. A survey of substance use by health care professionals and their attitudes to substance misuse patients (NHS staff survey). Journal of Substance Use 2008; 13(1):57-69.

**Richmond 1994**

Richmond RL, Anderson P. Research in general practice for smokers and excessive drinkers in Australia and the UK: III. Dissemination of interventions. Addiction 1994; 89(1):49-62.

**Rieckmann 2010**

Rieckmann T, Fuller BE, Saedi GA, McCarty D. Adoption of practice guidelines and assessment tools in substance abuse treatment. Subst Abuse Treat Prev Policy 2010; 5:4.

**Rivers 1998**

Rivers JE. Services for substance abusers in a changing health care system. American Behavioral Scientist 1998; 41(8):1136-1156.

**Roberts 2008**

Roberts LW, Johnson ME, Brems C, Warner TD. When providers and patients come from different backgrounds: perceived value of additional training on ethical care practices. TRANSCULT PSYCHIATRY 2008; 45(4):553-565.

**Robertson 2015**

Robertson A, Walker C, Stovall M, McCluskey L. Use of evidence-based substance use treatment practices in Mississippi. Eval Program Plann 2015; 52:198-204.

**Roche 1991**

Roche AM, Richard GP. Doctors' willingness to intervene in patients' drug and alcohol problems. Soc Sci Med 1991; 33(9):1053-1061.

**Roche 2001**

Roche AM, Watt K, Fischer J. General practitioners' views of home detoxification. Drug Alcohol Rev 2001; 20(4):395-406.

**Rohman 1987**

Rohman ME, Cleary PD, Warburg M, Delbanco TL, Aronson MD. The response of primary care physicians to problem drinkers. Am J Drug Alcohol Abuse 1987; 13(1-2):199-209.

**Ronzani 2005**

Ronzani TM, Ribeiro MS, Amaral MBd, Formigoni MLOdS. [Hazardous alcohol use: screening and brief intervention as routine practice in primary care]. Cad Saude Publica 2005; 21(3):852-861.

**Ronzani 2009**

Ronzani TM, Mota DCB, Souza ICWd. Alcohol prevention within primary care in municipalities in the state of Minas Gerais, Southeastern Brazil. Rev Saude Publica 2009; 43 Suppl 1:51-61.

**Rose 2016**

Rose GL, Badger GJ, Skelly JM, Ferraro TA, MacLean CD, Helzer JE. A Randomized Controlled Trial of IVR-Based Alcohol Brief Intervention to Promote Patient-Provider Communication in Primary Care. J Gen Intern Med 2016.

**Rosenstock 2010**

Rosenstock KIV, Jose das Neves M. [Nurse's role on basic health care regarding to the approach to drug addicted in Joao Pessoa, PB, Brazil]. Rev Bras Enferm 2010; 63(4):581-586.

**Rosso 1992**

Rosso S, Battista RN, Segnan N, Williams JI, Suissa S, Ponti A. Determinants of preventive practices of general practitioners in Torino, Italy. Am J Prev Med 1992; 8(6):339-344.

**Rosta 2003**

Rosta J. Determinants of physicians' prevention-related practice: a comparison between Aarhus, Denmark, and Mainz, Germany. Journal of Substance Use 2003; 8(1):42-46.

**Rowland 1989**

Rowland N, Maynard AK. Alcohol education for patients: some nurses need persuading. Nurse Educ Today 1989; 9(2):100-104.

**Rush 2013**

Rush B, McPherson-Doe C, Behrooz ReC, Cudmore A. Exploring core competencies for mental health and addictions work within a Family Health Team setting. Ment Health Fam Med 2013; 10(2):89-100.

**Saitz 2002**

Saitz R, Friedmann PD, Sullivan LM, Winter MR, Lloyd-Travaglini C, Moskowitz MA et al. Professional Satisfaction Experienced When Caring for Substance-abusing Patients: Faculty and Resident Physician Perspectives. J Gen Intern Med 2002; 17(5):373-376.

**Seppannen 2012**

Seppannen KK, Aalto M, Seppä K. Institutionalization of Brief Alcohol Intervention in Primary Health Care-The Finnish Case. Alcoholism: Clinical & Experimental Research 2012; 36(8):1456-1461.

**Sibthorpe 2002**

Sibthorpe BM, Bailie RS, Brady MA, Ball SA, Sumner-Dodd P, Hall WD. The demise of a planned randomised controlled trial in an urban Aboriginal medical service. Med J Aust 2002; 176(6):273-276.

**Skinner 2007**

Skinner N, Feather NT, Freeman T, Roche A. Stigma and discrimination in health-care provision to drug users: The role of values, affect, and deservingness judgments. Journal of Applied Social Psychology 2007; 37(1):163-186.

**Smith 2003**

Smith E, Mistral W. Shared care: Lessons from one model of shared care nursing in primary care. Drugs: Education, Prevention & Policy 2003; 10(3):263-270.

**Soares 2013**

Soares J, de Vargas D, Oliveira de Souza Formigoni ML. Knowledge and attitudes of nurses towards alcohol and related problems: the impact of an educational intervention. Revista da Escola de Enfermagem da USP 2013; 47(5):1172-1179.

**Spandorfer 1999**

Spandorfer JM, Israel Y, Turner BJ. Primary care physicians' views on screening and management of alcohol abuse: Inconsistencies with national guidelines. J FAM PRACT 1999; 48(11):899-902.

**Stockwell 1990**

Stockwell T, Bolt L, Milner I, Pugh P, Young I. Home detoxification for problem drinkers: acceptability to clients, relatives, general practitioners and outcome after 60 days. Br J Addict 1990; 85(1):61-70.

**Stoner 2014**

Stoner SA, Mikko AT, Carpenter KM. Web-based training for primary care providers on screening, brief intervention, and referral to treatment (SBIRT) for alcohol, tobacco, and other drugs. J Subst Abuse Treat 2014; 47:362-370.

**Strang 2007**

Strang J, Hunt C, Gerada C, Marsden J. What difference does training make? A randomized trial with waiting-list control of general practitioners seeking advanced training in drug misuse. Addiction 2007; 102(10):1637-1647.

**Strayer 2012**

Strayer SM, Pelletier SL, Rollins LK, Heim SW, Ingersoll KS, Ritterband LM et al. Evaluation of a Screening and Counseling Tool for Alcohol Misuse: A Virginia Practice Support and Research Network (VaPSRN) Trial. Journal of the American Board of Family Medicine 2012; 25(5):605-613.

**Tam 2013**

Tam M, Zwar N, Markham R. Australian general practitioner perceptions of the detection and screening of at-risk drinking, and the role of the AUDIT-C: a qualitative study. BMC Fam Pract 2013; 14(1):121-128.

**Taylor 2007**

Taylor P, Zaichkin J, Pilkey D, Leconte J, Johnson BK, Peterson AC. Prenatal screening for substance use and violence: findings from physician focus groups. Maternal & Child Health Journal 2007; 11(3):241-247.

**Thomas 2014**

Thomas K, Krevers B, Bendtsen P. Long-term impact of a real-world coordinated lifestyle promotion initiative in primary care: a quasi-experimental cross-sectional study. BMC Fam Pract 2014; 15:201.

**Thompson 2001**

Thompson J. Difficult behaviour in drug-misusing and non-drug-misusing patients in general practice--a comparison. British Journal of General Practice 2001; 51(466):391-393.

**Tober 1990**

Tober G, Raistrick D. Development of a district training strategy. Br J Addict 1990; 85(12):1563-1570.

**Tonnesen 2010**

Tonnesen H, Faurschou P, Ralov H, Molgaard-Nielsen D, Thomas G, Backer V. Risk reduction before surgery. The role of the primary care provider in preoperative smoking and alcohol cessation. BMC Health Serv Res 2010; 10:121.

**Townes 1994**

Townes PN, Harkley AL. Alcohol screening practices of primary care physicians in eastern North Carolina. Alcohol 1994; 11(6):489-492.

**Urada 2014**

Urada D, Teruya C, Gelberg L, Rawson R. Integration of substance use disorder services with primary care: health center surveys and qualitative interviews. Subst Abuse Treat Prev Policy 2014; 9:15.

**Vadlamudi 2008**

Vadlamudi RS, Adams S, Hogan B, Wu T, Wahid Z. Nurses' attitudes, beliefs and confidence levels regarding care for those who abuse alcohol: impact of educational intervention. NURSE EDUC PRACT 2008; 8(4):290-298.

**van Boekel 2014**

van Boekel LC, Brouwers EPM, van Weeghel J, Garretsen HFL. Healthcare professionals' regard towards working with patients with substance use disorders: Comparison of primary care, general psychiatry and specialist addiction services. Drug & Alcohol Dependence 2014; 134:92-98.

**van Boekel 2015**

van Boekel LC, Brouwers EPM, van Weeghel J, Garretsen HFL. Comparing stigmatising attitudes towards people with substance use disorders between the general public, GPs, mental health and addiction specialists and clients. International Journal of Social Psychiatry 2015; 61(6):539-549.

**Vargas 2008**

Vargas D, Luis MAnV. Alcohol, alcoholism and alcohol addicts: Conceptions and attitudes of nurses from district basic health centers. Rev Lat Am Enfermagem 2008; 16(4):543-550.

**Vargas 2010**

Vargas D, Oliveira M, Luís M. Care of alcoholic persons in primary care services: perceptions and actions of registered nurses. Acta Paulista de Enfermagem 2010; 23(1):73-79.

**Wallston 1976**

Wallston KA, Wallston BS, DeVellis BM. Effect of a negative stereotype on nurses' attitudes toward an alcoholic patient. J stud alcohol 1976; 37(5):659-665.

**Waring 1975**

Waring ML. The impact of specialized training in alcoholism on management-level professionals. J stud alcohol 1975; 36(3):406-415.

**Weinehall 2014**

Weinehall L, Johansson H, Sorensen J, Jerden L, May J, Jenkins P. Counseling on lifestyle habits in the United States and Sweden: a report comparing primary care health professionals' perspectives on lifestyle counseling in terms of scope, importance and competence. BMC Fam Pract 2014; 15:83.

**Whiteford 2015**

Whiteford M, Byrne P. Talking about alcohol: communities of practice and patient pathways. Journal of Research in Nursing 2015; 20(1):12-25.

**Williams 1999**

Williams K. Attitudes of mental health professionals to co-morbidity between mental health problems and substance misuse. Journal of Mental Health 1999; 8(6):605-613.
